# Factors influencing the timeliness of reporting highly pathogenic avian influenza outbreaks in Europe

**DOI:** 10.1371/journal.pone.0360917

**Published:** 2026-11-10

**Authors:** Gauthier Vercher, David R. J. Pleydell, Lina Awada, Paolo Tizzani, Julien Cappelle, Renaud Lancelot, Elena Arsevska, Carlène Trevennec

**Affiliations:** 1 Joint Research Unit Animal, Health, Territories, Risks, Ecosystems (UMR ASTRE), French Agricultural Research Centre for International Development (CIRAD), Montpellier, France; 2 Joint Research Unit Animal, Health, Territories, Risks, Ecosystems (UMR ASTRE), National Research Institute for Agriculture, Food and Environment (INRAE), Montpellier, France; 3 Joint Research Unit Animal, Health, Territories, Risks, Ecosystems (UMR ASTRE), University of Montpellier, Montpellier, France; 4 World Animal Health Information and Analysis Department (WAHIAD), World Organisation for Animal Health, WOAH, Paris, France; 5 Joint Research Unit Animal, Health, Territories, Risks, Ecosystems (UMR ASTRE), French Agricultural Research Centre for International Development (CIRAD), Sainte Clotilde, La Réunion, France; Federal Ministry of Agriculture and Rural Development, NIGERIA

## Abstract

Timely reporting of zoonotic infectious diseases allows for rapid response and mitigation by health authorities. However, little is known about the factors influencing the timeliness of disease outbreak reports. The World Animal Health Information System provides extensive data on delays that arise between first suspecting and subsequently reporting outbreaks of notifiable animal diseases. Here we analysed these report delays using a Bayesian semi-parametric survival model, to assess socio-demographic, epidemiological and cultural covariates on the timeliness of disease outbreak reports in Europe, using highly pathogenic avian influenza as a model disease. The survival model included time-varying coefficients and a penalized spline to account for temporal fluctuations in reporting rates, and parsimonious shrinkage (Bayesian model selection) was obtained using Dirichlet-Laplace priors. The results indicated that outbreaks occurring in wild birds, high outbreak notification rates, country, day of week and national holidays were all associated with delayed outbreak reporting times. Higher political stability and better corruption control, as defined by the World Bank, were also associated with longer reporting times, while high per capita gross national income and local poultry density were associated with shorter reporting delays. Better knowledge of surveillance capacities and modalities on a smaller scale could help further understand the reasons for the associations observed between these factors and reporting delays. Our results can help disease surveillance teams improve predictions of the day-to-day dynamics of epizootics and improve disease-introduction risk assessments. These results may also help inform tailored, country-specific recommendations for health authorities, delivered confidentially through international health organizations, to improve disease reporting.

## Introduction

Zoonotic infectious diseases (ZIDs) threaten animal and human health. Effective surveillance, prevention and control of ZIDs represent ambitious milestones for national health authorities due to the diverse hosts involved and the complex eco-epidemiology typical of most zoonotic pathogens [1]. Animal husbandry is undergoing major shifts. Production systems are becoming more intensive, and livestock farms are increasingly close to wild bird habitats, and, as a result, contacts between humans, livestock, and wildlife are also intensifying [2]. At the same time, cross-border trade and travel are increasing. These combined trends require countries to adopt a holistic approach to ZID management [3]. Numerous epidemics of zoonotic infectious diseases (ZIDs) have highlighted the importance of efficient surveillance systems. Notable examples include the extensive spread of highly pathogenic avian influenza (HPAI) in Europe in 2022 [4] and the recent coronavirus pandemic caused by SARS-CoV-2 [5]. These events underscore the need for surveillance schemes capable of rapidly detecting emergence events and ensuring transparent data sharing with relevant national and international health authorities within a One Health framework [1,6].

Rapid detection and reporting of ZID outbreaks allow timely responses and mitigation measures [7]. Notifiable disease surveillance systems are deployed nationally [8] to collect detailed outbreak information; however, these systems often rely on data from multiple sources, and there are time delays between observing disease symptoms, sending samples to a laboratory, testing and reporting to international organisations [9]. These delays can lead to inefficiencies and sub-optimal performance in early-warning systems [10].

While various studies have explored factors influencing the timeliness of disease reporting, most have focused on global trends and human health rather than animal diseases in Europe [6]. The World Organisation for Animal Health (WOAH) estimates a median reporting delay of 11 days, with both the confirmation and notification processes of 85 listed diseases across the globe contributing to these delays [11]. The analysis of the World Animal Health Information System (WAHIS) [12] data, the global animal health reference database of the WOAH, has highlighted several trends that suggest various factors can influence report delays. Economic status, regional differences and disease frequency are known to affect reporting rates [11]. Similarly, electronic communication, surveillance capacity, and institutional preparedness also play crucial roles [13,14].

Oppenheim *et al*. [15] created the Epidemic Preparedness Index (EPI), an indicator for which the preparedness component was evaluated using the timeliness of reporting human influenza cases, and further underscores the impact of public health infrastructure, economic resources, and governance on reporting efficiency [15]. This index was subdivided into five sub-indexes – public health infrastructure; physical infrastructure; institutional capacity; economic resources; and public health communication, which further highlights the perceived importance of various socio-economic factors on the timeliness of disease reporting.

While these findings provide valuable insights, they are primarily derived from public health systems, and comparable large-scale evaluations in animal health, particularly using WAHIS data, remain limited

This study assessed the factors influencing timeliness of reporting of highly pathogenic avian influenza (HPAI) outbreaks to the WOAH. HPAI provides a prime example of an infectious disease that exhibits a rapidly increasing incidence in Europe [11]. It is a highly contagious viral disease, mainly affecting domestic and wild birds. It has also demonstrated transmission to marine and terrestrial mammal populations [16,17]. This zoonotic virus has been the source of worldwide outbreaks, leading to several million deaths in wild birds, culled poultry, and sporadic human cases. As a WOAH-listed disease, new HPAI outbreaks that are considered as exceptional epidemiological events must be reported through WAHIS within 24 hours after laboratory confirmation. In this context and for the purpose of this study, timeliness refers to the time required for outbreak information to become available in WAHIS after the detection of the event. The reporting obligations and criteria for what constitutes an exceptional epidemiological event are defined in the Terrestrial Animal Health Code, which sets international standards for animal disease surveillance and notification [18]. Using this disease as a case study allows inference and insights from a large quantity of outbreak data notified by well-established national surveillance systems in many European countries [11,19]. The results can help identify domains for improvement in current animal health surveillance systems, particularly in the detection, confirmation, and response to infectious disease outbreaks.

## Materials and methods

### HPAI report delay data

According to WOAH regulations, members are required to submit immediate notification (IN) reports, through WAHIS, within 24h of any new event of a WOAH listed-disease, where an event corresponds to an unusual epidemiological event as described in the Terrestrial Code Chapter 1.1 Article 1.1.3 [18]. These immediate notification (IN) reports, which signal disease emergence events, should be followed-up by weekly reports that provide further information on the evolution of the event [18]. These follow-up reports (FUR) should continue until the listed disease has been eliminated or the situation is considered sufficiently stable, at the discretion of the Member Country. At that point, member countries may submit reports every 6 months, providing periodic updates on the disease status and fulfilling reporting obligations. The FURs contain all outbreaks included in the IN (which could be updated) and any subsequent outbreaks related to the event [18]. This can result in the duplication of outbreak information within FURs, which is represented graphically in Fig 1. For each outbreak, the IN and FUR include the observed start date and the first reporting date. The laboratory confirmation date is given only once and corresponds to the first testing declared in the IN report. Thus, the confirmation date for subsequent outbreaks is not provided in FURs.

**Fig 1:**
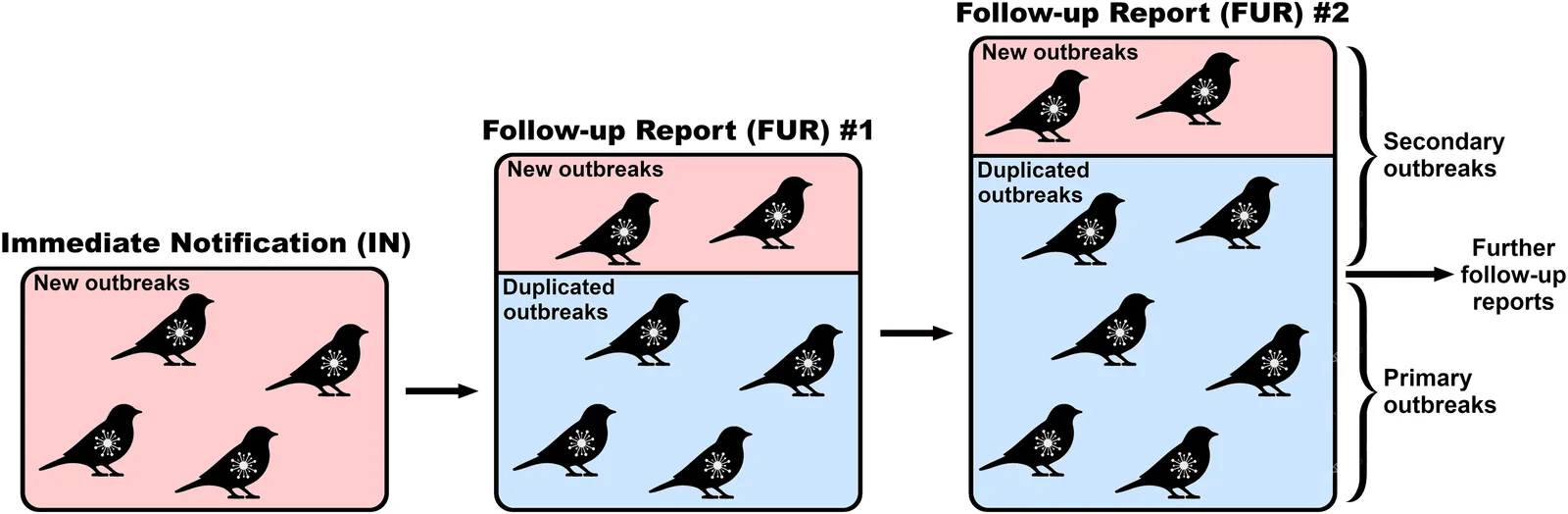
Structure of disease outbreak information in the World Animal Health Information System (WAHIS). Information related to HPAI primary outbreaks declared in an immediate notification (IN) report is duplicated in a series of subsequent weekly follow-up reports (FUR), where it is complemented by similar information on all known secondary outbreaks. All such duplicates in FURs must be filtered out prior to statistical analysis. In the above figure, outbreak reports in the red boxes provide new information about an ongoing epidemic, as opposed to the outbreaks in the blue boxes, which were also documented in previous reports.

This study focused on the outbreak level, i.e., individual disease occurrences in defined epidemiological units, rather than the broader ‘event’ scale, which, according to the WOAH, may consist of a single outbreak or a series of epidemiologically‑related outbreaks from the first notification through to the final report. A comprehensive data set was retrieved from WAHIS [12] on the 30th of May 2024. The spatio-temporal focus was on Europe between January 1st 2005 and December 31st 2023. Countries have been anonymized to avoid stigmatization and discrimination. The data were filtered to include only reports in which the disease was classified as “Highly Pathogenic Avian Influenza (HPAI) in poultry” or “Influenza A viruses of high pathogenicity (non-poultry including wild birds) (2017-)”. Reports involving non-bird species were excluded. The data curation process is described in the supplementary materials S1 in S1 File. Unique outbreaks were filtered based on the outbreak identifier, to avoid duplicates arising in the WAHIS data when either (1) outbreaks include multiple species, or more commonly, (2) outbreaks are re-reported in FURs (Fig 1). For each outbreak, we kept the earliest reporting date. The final data set included unique outbreaks, for which we had the outbreak start date, the date of confirmation by a reference laboratory, the date the outbreak was first reported on WAHIS, the country and the type of report (IN or FUR) in which the outbreak was first reported. Outbreaks that first appeared in IN reports were referred to as “primary outbreaks”. Outbreaks that first appear in FURs were referred to as “secondary outbreaks”.

The primary metric of interest in the current study was the report delay, defined as the difference in days between the *Outbreak_start_date* and the *Report_date*. Fig 2 provides a graphical description of the various dates and delays associated with each outbreak report. Observations with inconsistent values for the reporting delay, such as negative values, were excluded from the dataset.

**Fig 2:**
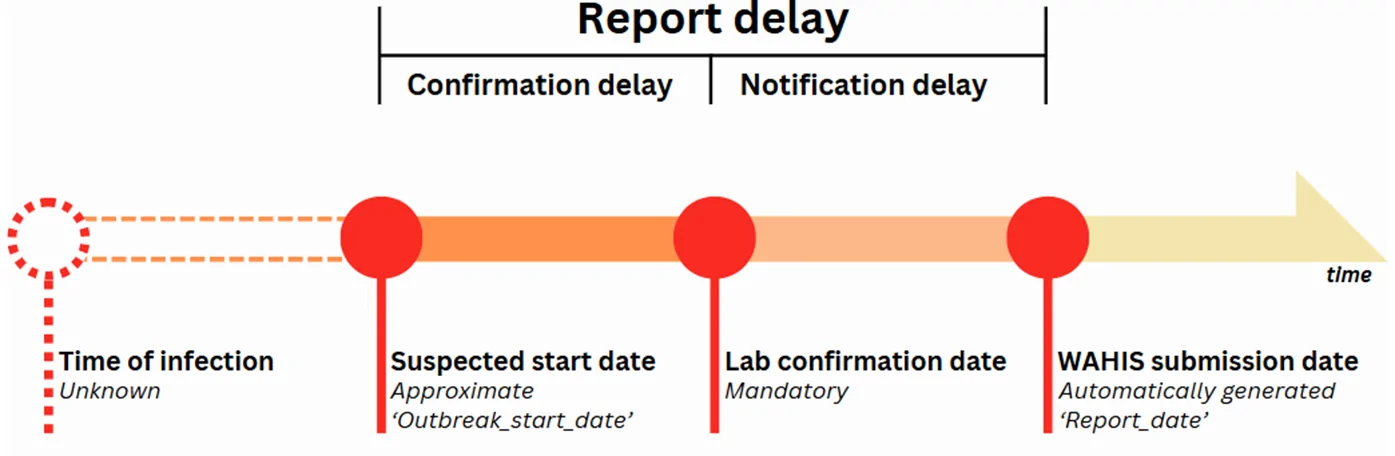
Confirmation, notification, and reporting delays. Quoted elements are the names of covariates in the WAHIS database. Declarations of confirmation date are only mandatory for primary outbreaks, and are typically missing from WAHIS data for secondary outbreaks.

### Covariate selection

Covariates were selected based on epidemiological and socio-economic indicators used in previous works to evaluate the timeliness of disease reporting [11,15]. We also included additional factors that could impact the effectiveness of the reporting system, such as the current incidence of the disease in a given country and days off, comprising of national holidays and weekends. To account for potential confounding between correlated predictors (e.g., socio-economic indicators), we assessed multicollinearity using cross-correlation analysis and removed highly correlated variables (Pearson’s |*r*| > 0.8), retaining the most relevant and stable indicators. A subsequent Variance Inflation Factor (VIF) [20] analysis was conducted to ensure that no remaining covariates exhibited problematic multicollinearity. Detailed information on these covariates, including their sources and descriptions, is provided in Table 1.

**Table 1:** Candidate covariates considered for the statistical analysis of HPAI report delays in Europe. Covariates in bold and annotated with an asterisk (*) indicate covariates which were eventually included in a Bayesian semi-parametric survival model. Covariates in the regular text were filtered out during the data curation processes described in the supplementary material section in S1 File.

Covariate	Description	Type	Source
Country*	Country in which the outbreak was reported	Factor	WAHIS reports
GDP	Gross domestic product at the time of the report	Numeric	World Bank World Development Indicators
GNI*	Gross national income at the time of the report	Numeric	World Bank World Development Indicators
Stability Score*	Government stability and capacity under stress	Numeric	World Bank Worldwide Governance Indicators
Control of corruption Score*	Public sector corruption	Numeric	World Bank Worldwide Governance Indicators
Internet Usage*	Internet users per 100 people	Numeric	World Bank World Development Indicators
Phone Usage*	Number of phone line subscriptions per 100 people	Numeric	World Bank World Development Indicators
State Structure	Indicates whether the country has federal or centralized governance	Categorical	[21]
Wild*	Indicates whether the outbreak occurred in domestic (0) or wild (1) animals	Categorical	WAHIS reports
Nearby Poultry Density*	Dasymetric poultry density (chickens + ducks) extracted at outbreak coordinates from a 5 arc-minute grid	Numeric	[22]
Nearby Chicken Density	Dasymetric chicken density extracted at outbreak coordinates from a 5 arc-minute grid	Numeric	[22]
Nearby Duck Density	Dasymetric duck density extracted at outbreak coordinates from a 5 arc-minute grid	Numeric	[22]
Veterinarian per Capita	Number of veterinarians per capita in the country	Numeric	WAHIS Animal Health Capacities data
Incidence of Recent Outbreaks*	Total number of outbreak reports in the same country in the 4 weeks prior to the given outbreak report	Numeric	WAHIS reports, personal production
Follow-up Report (FUR)*	Indicates whether the outbreak was first reported in an immediate notification (IN) (0) or a follow-up report (FUR) (1)	Categorical	WAHIS reports
Years since First HPAI Case	Number of years since the first recorded HPAI case in a given country	Numeric	WAHIS reports
Day of week*	The sequence of weekdays between the suspicion and submission dates submitted	Categorical	WAHIS reports
Holidays*	Indicates if a national holiday occurs within the report delay	Categorical	WAHIS reports, Nager.Date API (https://date.nager.at/api/v3/PublicHolidays/)

### Survival analysis

Statistical investigation of the HPAI report delays was performed using survival analysis – a branch of statistics dedicated to the analysis of time-to-event data [23]. Here we use non-parametric methods for data visualization and Bayesian semi-parametric methods for model fitting and inference. The semi-parametric approach provided a suitable regression framework for inferring the effects of multiple covariates and time-varying effects. The choice of the Bayesian approach provided a practical paradigm for quantifying parameter uncertainty and model selection. Technical details of the implementation of these methods are provided below.

One particularity of survival analysis is the requirement to account for uncertainties arising from data censoring. Here, report delays were calculated as the difference between two dates – they were thus measured in days and provided no information as to when, within the given day an outbreak report was submitted. In other words, the report delays were *interval censored*, and our analyses were adapted accordingly. Moreover, since the duration of the censor interval was constant (24 hours), we effectively performed a discrete time survival analysis.

### Non-parametric survival curves

Non-parametric (i.e., data driven) *survival curves*, describing how the probability to have not yet reported an outbreak drops from one to zero with time, were generated using Turnball’s non-parametric maximum likelihood estimator for interval censored data [24]. These non-parametric survival curves were generated globally (using all data), and separately for each country using the corresponding country-specific subset of the data. The non-parametric survival curves were used for: (1) visual representation of the distribution of report delays, and (2) for assessing the survival curves generated from the semi-parametric survival model (described below).

### Semi-parametric survival model

Two central components of survival analysis are the survival function, *S*(*t*), which gives the probability that an event has not occurred by time *t*, and the hazard function, *h*(*t*), which describes the occurrence rate of events at time *t* [25]. These are related as follows


$$\begin{array}{c} S(t)=p(T>t)=\exp(-\int_{0}^{t}h(u)du) \end{array}$$ (1)

where *T* is a continuous random variable depicting the time to an event. Here, the event in question is the submission of an outbreak report, and *T* is the delay (in continuous time) between the time an outbreak was first suspected and the submission of the outbreak report.

Let *Y* be a random variable representing a report delay in discrete time, with *y* its observed value. Since *Y* is the difference between suspicion and submission dates it is measured in days and takes integer values only. A value of *y* = 0 would imply that the initial suspicion, and the sampling, testing and reporting, all occurred on the same day (which never occurs in practice in the data). The likelihood of a report delay of *y* days is


$$\begin{array}{cc} p(Y=y) & =p(y<T\leq y+1)\\ & =S(y)-S(y+1) \end{array}$$ (2)

where *S*(*t*), the survival probability at time *t*, accounts for temporal changes in the hazard, *h*(*t*).

Preliminary analyses suggested that a parametric (Weibull) proportional hazards model was insufficiently flexible to characterize temporal changes in the baseline hazard function. Thus, we developed a semi-parametric survival model, which consisted of a piece-wise exponential model where the hazard function could vary in daily time-steps – thus providing a flexible framework for modeling temporal changes in reporting rates. Our discrete time survival function was defined for any integer *t* > 0 as follows:


$$\begin{array}{cc} S(t) & =\exp(-\sum_{d=0}^{t-1}h(d))\\ & =S(t-1)\times \exp(-h(t-1)) \end{array}$$ (3)

where *S*(0) = 1 and *h*(*d*) models the reporting rate *d* days after the day on which a given outbreak was first suspected.

### Time-varying hazard function

Survival models are often defined in terms of a hazard function of the form


$$\begin{array}{c} h(t)=h_{0}(t)e^{\beta_{1}x_{1}+\beta_{2}x_{2}+...+\beta_{m}x_{m}} \end{array}$$ (4)

where *h*_0_ is a baseline hazard function, *m* is the number of covariates, $x_{i}$ is the $i^{th}$ covariate and $\beta_{i}$ is its regression coefficient. Often the regression part of the hazard is assumed to remain constant in time and simply scales (proportionally) the baseline hazard. However in our study, two cultural factors – day of week and public holidays (Table 1) – could potentially cause the hazard to change from day to day. An additional time-varying factor was added to indicate if *d* = 0, i.e., to reduce the hazard on the day that the outbreak was first suspected (omitted in Table 1). A penalized smoothing spline (P-spline) [26,27] was used to model the baseline hazard rate as a function of time. Our discrete time hazard function was thus


$$\begin{array}{c} h(t)=\exp\left(\beta_{0}+\sum_{l=1}^{k}B_{l}(t)\beta_{ps,l}\right)\exp\left(\sum_{j=1}^{m}x_{j}(t)\beta_{j}\right) \end{array}$$ (5)

where $B_{l}(t)$ is the $l^{th}$ spline basis function evaluated at time *t*, $\beta_{ps,l}$ is its coefficient, $\beta_{0}$ is an intercept representing the baseline log-hazard and $x_{j}(t)$ represents *t*he $j^{th}$ covariate which may vary in time. Covariates for the weekday and holiday effects were included as indicator variables, allowing the hazard to vary from day to day. These covariates provided an efficient means to characterize the effects of operational delays in reporting arising from institutional working schedules.

The P-spline was defined using 50 internal knots and degree-3 polynomials, with knots spread evenly over the range of *y*^1/4^. The fourth root transform was used to ensure greater knot density (and therefore flexibility) over the lower (and more abundant) values of the report delays (*y*). To limit over-fitting, a second-order difference penalty was applied to the spline coefficients. This is a common practice in the Bayesian P-spline literature [26,28] and provides a suitable constraint against overfitting for models with a large number of knots.

### Bayesian inference

A Bayesian approach was used to estimate the parameters of the semi-parameteric regression model. The prior distributions used for our model are:


$$\begin{array}{cc} \beta_{0} & \sim \text{Normal}(\text{mean}=0,\text{st.dev}=1000)\\ \beta_{j} & \sim \text{Laplace}(\text{location}=0,\text{scale}=\phi_{j})\\ \phi_{j} & \sim \text{Gamma}(\text{shape}=\frac{1}{2},\text{rate}=\frac{1}{2})\\ \beta_{ps} & \sim \text{MVNormal}(0,\tau S) \end{array}$$

where j∈[1,m] is an index for a given regression coefficient. The combination of a Laplace prior for $\beta_{j}$ and a gamma prior for $\phi_{j}$ gives a Dirichlet-Laplace prior (equation 10 in [29]) – this prior provides’Bayesian shrinkage’ which helps maintain parsimony in highly parameterised regression models since it pushes coefficients towards zero unless there is sufficient evidence to justify other values. $\beta_{ps}$ is the vector of P-spline coefficients, τS provides the Cholesky decomposition of the precision matrix, τ is a scaling factor that controls smoothing and *S* is the square of a second-order difference matrix (adjusted to avoid squaring at the final knot) which provides a penalty against large second-order differences in $\beta_{ps}$. Following several trial runs we set τ (a penalty coefficient) to 50, providing a large degree of flexibility without visible evidence of over-fitting.

According to Bayes law, the posterior likelihood of the parameters given the data is proportional to the product of the likelihood of the data given the parameters multiplied by the prior likelihood, i.e.,


$$\begin{array}{c} \pi (\beta ,\beta_{ps},\phi |y,X,S,\tau )\propto \pi (y|\beta ,\beta_{ps},X)\pi (\beta |\phi )\pi (\beta_{ps}|S,\tau )\pi (\phi ) \end{array}$$ (6)

where $y=(y_{1},...,y_{n})$ is the vector of response delays, *X* is the n×m matrix of (both constant and time varying) covariates, $\beta =(\beta_{0},...,\beta_{m})$ is the set of regression coefficients, $\beta_{ps}=(\beta_{\text{ps,1}},...,\beta_{\text{ps,k}})$ is the set of P-spline coefficients, and $\phi =(\phi_{1},...,\phi_{m})$ is the set of scale parameters for the Dirichlet-Laplace priors. The expression of the likelihood is


$$\begin{array}{c} \pi (y|\beta ,\beta_{ps},X)=\prod_{i=1}^{n}\pi (y_{i}|\beta ,\beta_{ps},X_{i}) \end{array}$$

where


$$\begin{array}{cc} \pi (y_{i}|\beta ,\beta_{ps},X_{i}) & =p(y_{i}<T_{i}\leq y_{i}+1)\\ & =S(y_{i}|\beta ,\beta_{ps},X_{i})-S(y_{i}+1|\beta ,\beta_{ps},X_{i}) \end{array}$$

as in equation (1) above.

### Markov chain Monte Carlo

Bayesian inference was performed using a Markov Chain Monte Carlo (MCMC) technique known as Adaptive Parallel Tempering (APT) [30,31]. The APT algorithm helps navigate complex, and possibly multi-modal, posterior distributions by running multiple chains in parallel at different temperatures. At temperature $T_{\kappa }$ the tempered posterior likelihood is:


$$\begin{array}{c} \pi_{\kappa }(\theta_{\kappa }|y)\propto \pi (\theta_{\kappa }|y{)}^{1/T_{\kappa }}, \end{array}$$

where $\pi (\theta_{\kappa }|y)$ is the posterior likelihood of the $\kappa^{th}$ parameter set $\theta_{\kappa }$ given the data *y* and $T_{\kappa }$ is the temperature associated with the $\kappa^{th}$ chain in the “temperature ladder” (the ordered set of temperatures being used). Higher temperatures provide flatter likelihoods which facilitate larger MCMC jumps. Additional jump rules permit switching parameter sets between adjacent chains in the temperature ladder. Inference is based solely on samples obtained from the coolest chain where *T*_1_ = 1, which leaves the target likelihood unmodified. All other temperatures are adapted to facilitate efficient mixing. We performed APT with 15 different temperatures – providing a practical compromise between computation time and improved mixing efficiency. At each temperature adaptive Metropolis-Hastings algorithm [32,33] was used to provide block updates of all parameters. The APT algorithm was run eight times for 1,111,111 iterations with thinning set at 100. Following the removal of a burn-in the final 6250 samples were retained for each of the eight runs, giving a total of 50000 samples for performing posterior analyses. The effective sample size (ESS) of each parameter in the MCMC output was assessed using the coda::effectiveSize. The choices described above resulted in the ESS of every parameter being greater than 1000 (a threshold that we considered sufficiently large for inference), the minimum ESS was 1135 and the maximum was 4363.

### Implementation

All analyses were performed using the statistical language R [34]. Non-parametric survival functions were estimated using the survival package [35]. The Bayesian semi-parametric survival model was implemented in NIMBLE [36]. The P-spline was generated using function ps in package dlnm [37]. The package nimbleNoBounds [38] was used to assist with MCMC efficiency. The APT algorithm was performed using nimbleAPT [39]. MCMC convergence diagnostics were performed using coda [40]. The packages forestplot [41] and ggplot2 [42] were used for plotting results.

## Results

### HPAI report delay data

The initial data set consisted of 19,855 HPAI outbreaks reported throughout Europe. After filtering out 14 outbreak reports with negative report delays, duplicated outbreaks appearing in FURs, and any outbreaks for which covariates were missing, the final data set provided a comprehensive overview of 12,166 outbreaks during the period 17/01/2006–02/05/2023 (Figs 3 and S1 in S1 File).

**Fig 3:**
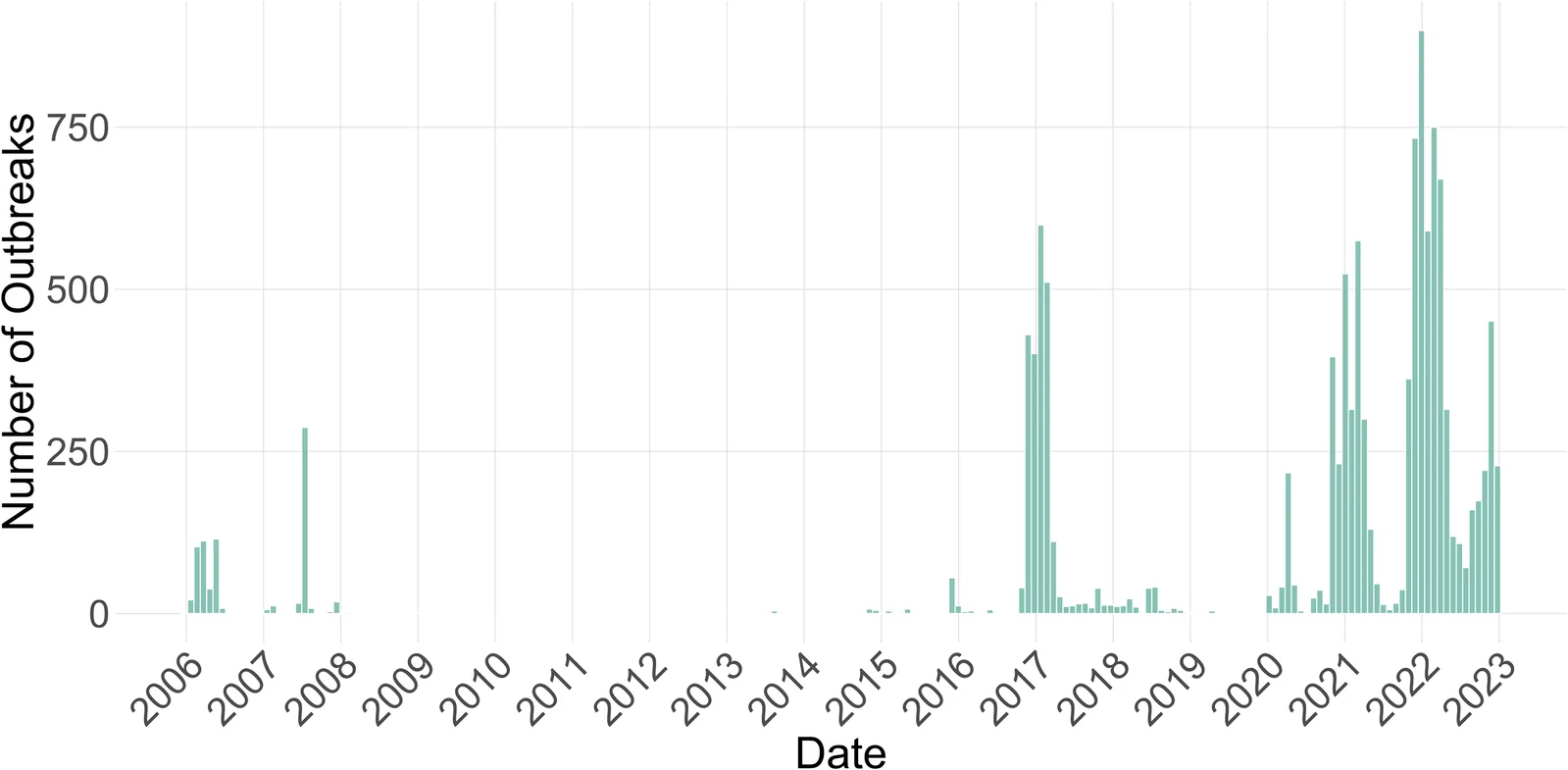
Number of Highly Pathogenic Avian Influenza outbreaks in domestic and wild birds of Europe reported in the World Animal Health Information System (WAHIS) between January 2006 and May 2023.

Among the 53 WOAH member countries in the Europe region (according to WAHIS definition, which includes but is not limited to the European Union), we found 32 countries reporting HPAI outbreaks within the study period. Most outbreaks were reported in France, Germany, and Hungary, with 3165, 2619 and 1018 outbreaks reported, respectively. The countries reporting the least outbreaks included Finland, Bosnia-Herzegovina and Luxembourg, with two, five and six outbreaks reported, respectively.

### Covariate selection

After evaluation of collinearity (Fig. S2 in S1 File), and the variance inflation factor (VIF) (Fig. S3 in S1 File), the final set of covariates retained for further analysis included a mix of epidemiological, socio-economic and environmental factors. These covariates are highlighted in Table 1, and include: Country, Gross national income (GNI) per capita, Stability score, Control of corruption score, Internet usage, Phone usage, Wild, Local poultry density, Incidence over 4 weeks, and FUR.

### Temporal patterns in confirmation and submission dates

The frequency of confirmations and submissions on the various days of the week, for immediate notifications, are presented in Table S1 in S1 File. These data reveal that, among 590 total immediate notification reports, Friday was the most frequent confirmation day (132 confirmations) and Thursday the most frequent reporting day (130 reports). Among non-weekend days, the most frequent reporting day was consistently the day following the confirmation day, with the exception for confirmations on Fridays, where the most frequent reporting day is the same day. Sunday was the least active day for confirmations (seven instances), and Saturday was the least active day for reporting (32 instances). This descriptive analysis focuses on weekdays only, the influence of public holidays is incorporated separately in the Bayesian survival model of reporting delays.

### Non-parametric survival curves

A non-parametric survival curve, describing the proportion of unreported outbreaks as a function of time is presented in supplementary figure S4 in S1 File. Seven days after the suspected start of an outbreak 74% of outbreaks were still unreported, and the median reporting delay was 10 days. By days 19 and 45, 75% and 90% of outbreaks had been reported respectively. However, 1% of reports took over 163 days, 0.05% of reports took over one year, and one report took 604 days. Non-parametric survival curves also indicated large differences between countries in report timeliness (Fig 4). The maximum report delay per country was less than 31 days in 25% of countries, greater than 264 days in 25% of countries, and had a median of 98 days.

**Fig 4:**
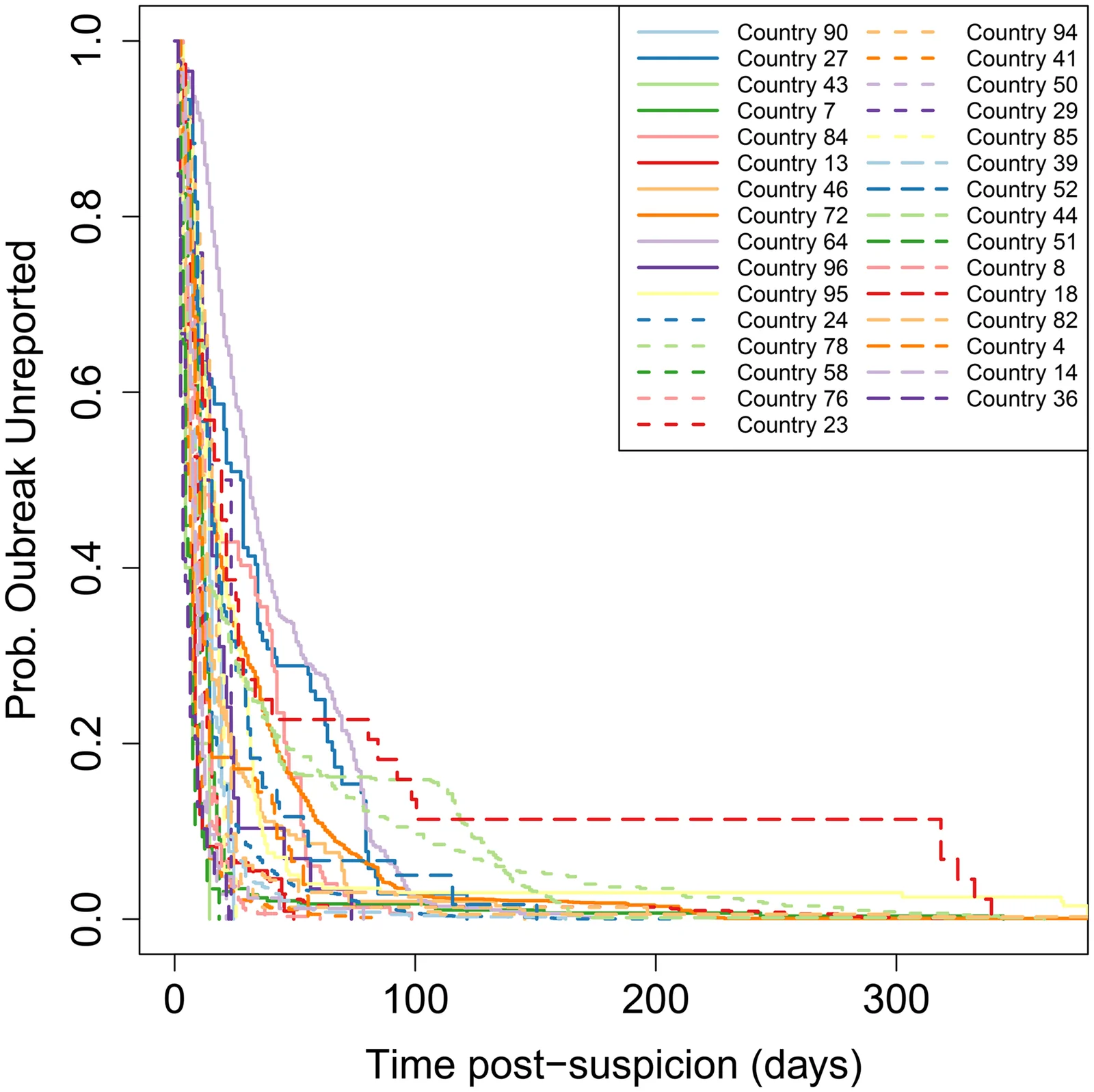
Between-country comparison of non-parametric survival curves describing how the probability that an outbreak remains unreported decreases with time.

### Model results

The survival curve generated by the semi-parametric model follows the non-parametric curve generated from the full dataset very closely (Fig. S5 in S1 File). The credibility intervals (CI) for the model-generated survival curves are larger than those of the non-parametric curves because the model accounts for between-country differences (compare Figs 4 and 7) whereas the non-parametric curve neglects these differences (Fig. S5 in S1 File).

### Parameter estimates and hazard ratios

The estimated mean and 95% CIs for the regression coefficients are provided in Table S2 in S1 File and plotted in Figs 5 and 6. Because the model uses a Bayesian shrinkage prior that pulls weak signals toward zero, coefficients whose 95% credible intervals do not include zero can be interpreted as having statistically supported associations with HPAI reporting delays. For categorical covariates, these coefficients represent the log of the hazard-ratio, which provides a measure of the relative risk of reporting an outbreak at any given time. For comparison purposes, the posterior distribution of the hazard ratios calculated for the continuous (numeric) covariates is presented in Table S4 in S1 File.

**Fig 5:**
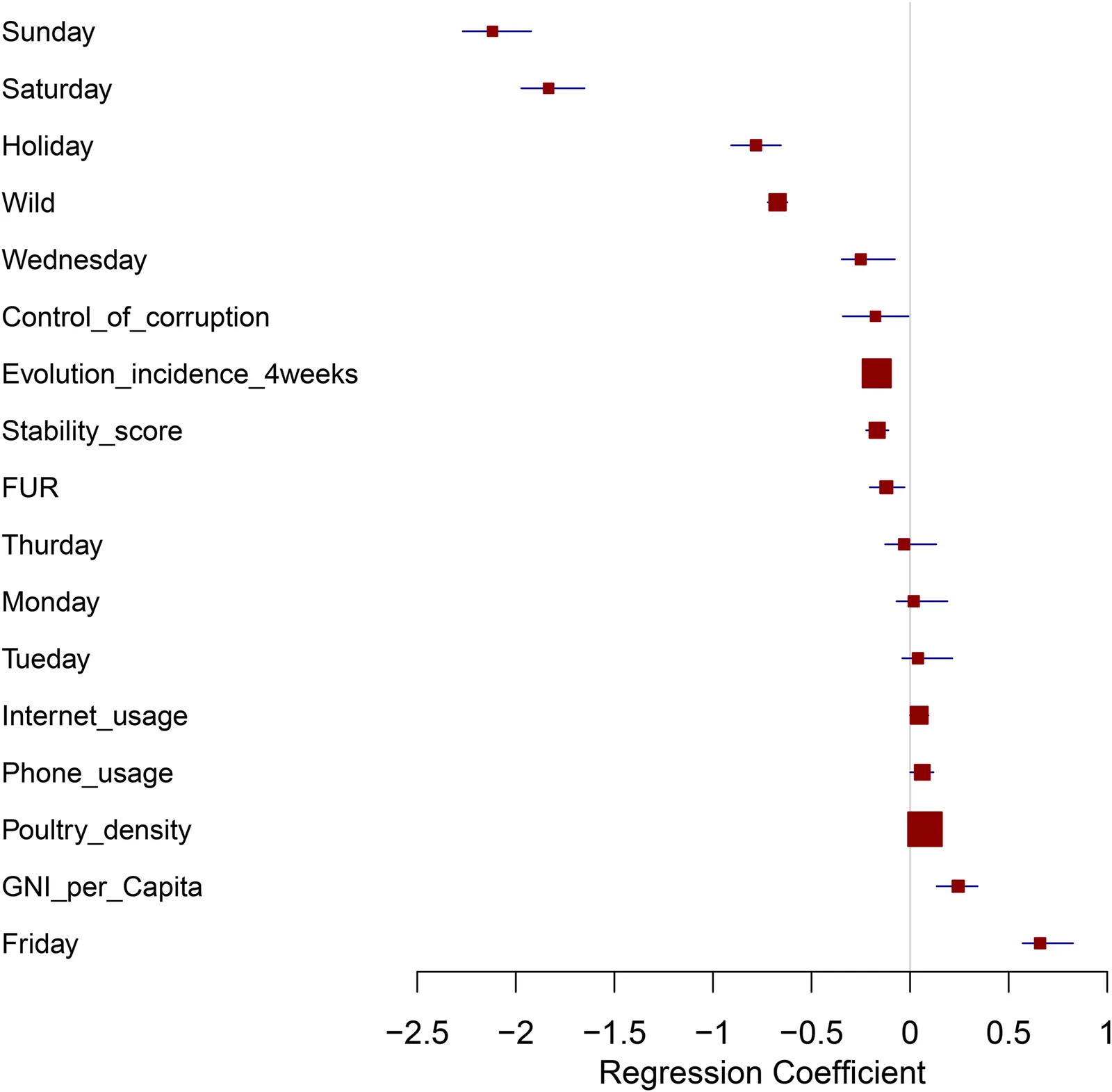
Uncertainty in the regression coefficients (β) associated with the various covariates used to model HPAI report delays in Europe. Red squares represent the posterior mean of each coefficient, and horizontal bars represent the 95% credibility intervals (CIs). The size of the red squares is proportional to the relative posterior density evaluated at the posterior mean, so coefficients with narrower CIs appear with larger squares. Positive and negative coefficients increase and decrease reporting rates respectively. The factor day zero (d = 0) is omitted to preserve readability. Numerical values are presented in Table S2 in S1 File.

**Fig 6:**
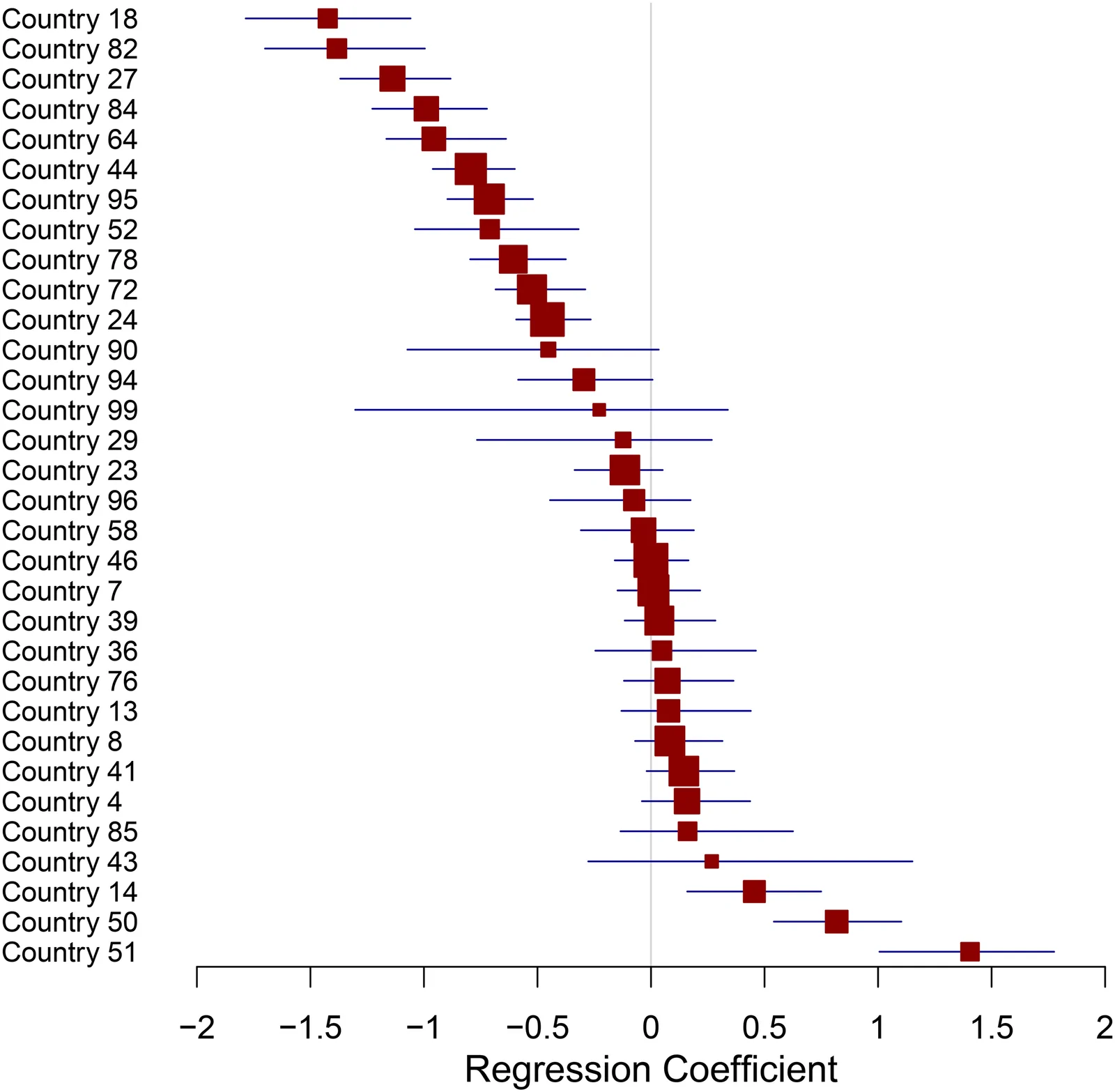
Estimated regression coefficients (log hazards ratios) associated with different countries included in the analyses of HPAI report delays. The red squares represent expected values. The bars represent the 95% CIs. Lower values are associated with longer report delays.

### Country effect

The mean and 95% CIs of the regression coefficients for each country are presented in Fig 6 and associated hazard ratios are presented in Table S3 in S1 File. Considerable between-country variation was detected, for example, the hazard ratio was 4.2 (95% CI: 2.7,5.9) in the fastest reporting country and 0.25 (95% CI: 0.17, 0.35) in one of the slowest reporting countries. This between-country variation is further illustrated by the expected reporting times (Figs 7 and 8, Table S6 in S1 File),which account for uncertainty in other model parameters as observed variation in the country-specific covariate data. These results emphasize that large variations in reporting times arise from country-specific differences, such that outbreak notifications might be reported up to eight times faster in some countries than in others (Table S6 in S1 File). Country-specific survival curves, which integrate all variation in covariate data observed at the country level, are presented in Fig 7.

**Fig 7:**
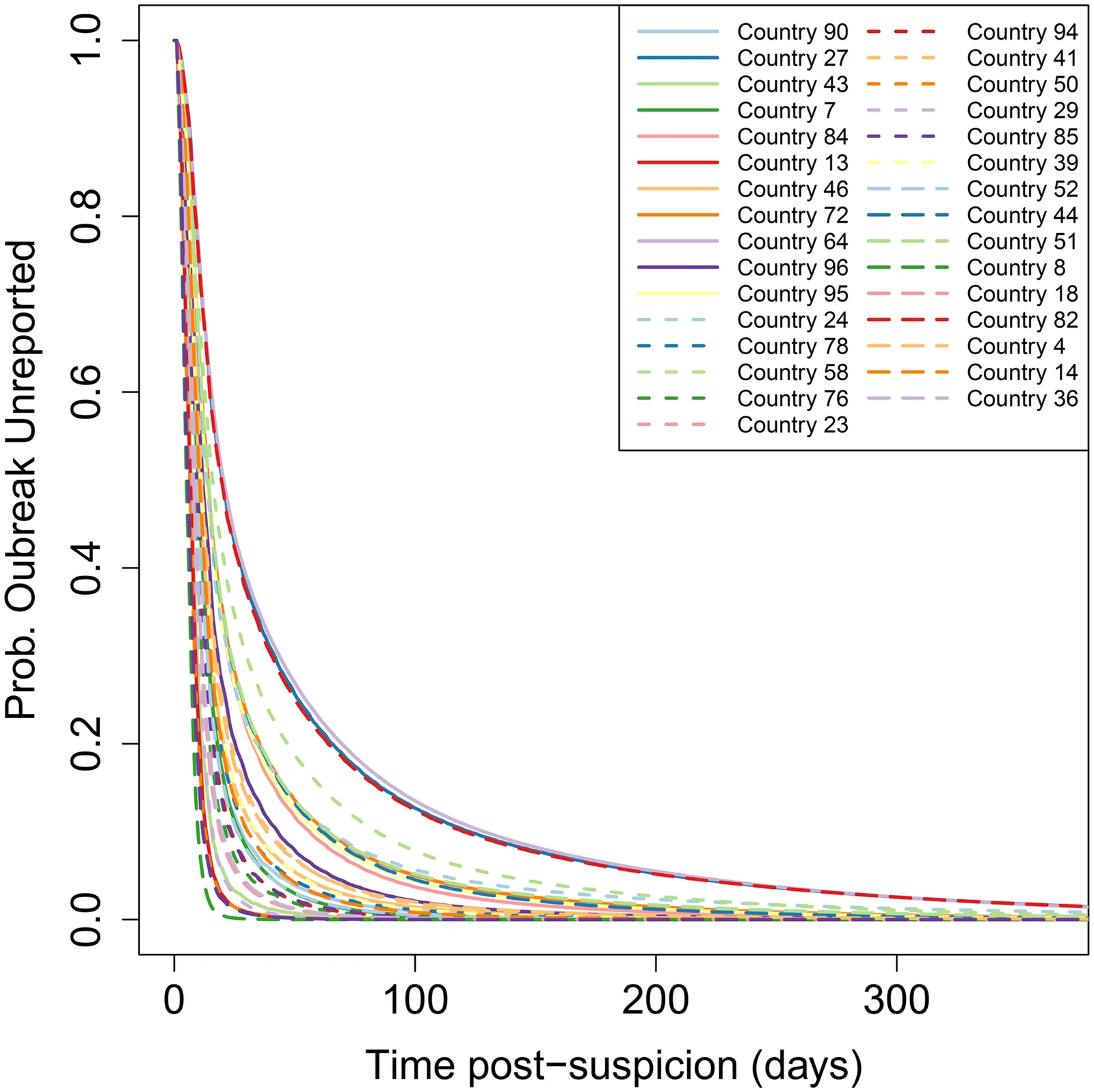
Between-country comparison of expected survival curves describing how the probability that an outbreak remains unreported decreases with time.

### Time varying effects

Three time-varying factors were included in our model: day of week, public holidays and day zero (d = 0). The expected values and 95% CIs of the log hazard ratios associated with these factors are presented in Fig 5 and Table S2 in S1 File. The day zero factor had the largest effect, with a hazard ratio of 0.032 (95% CI: 0.000,0.206). Hazard ratios varied over the working week and were lower on Sundays (0.12, 95% CI: 0.103, 0.146), Saturdays (0.16, 95% CI: 0.14, 0.19), national holidays (0.46, 95% CI: 0.40, 0.52) and Wednesdays (0.78, 95% CI: 0.70, 0.93). The hazard ratio was the highest on Fridays (1.94, 95% CI: 1.77, 2.29) (Fig 5).

**Fig 8:**
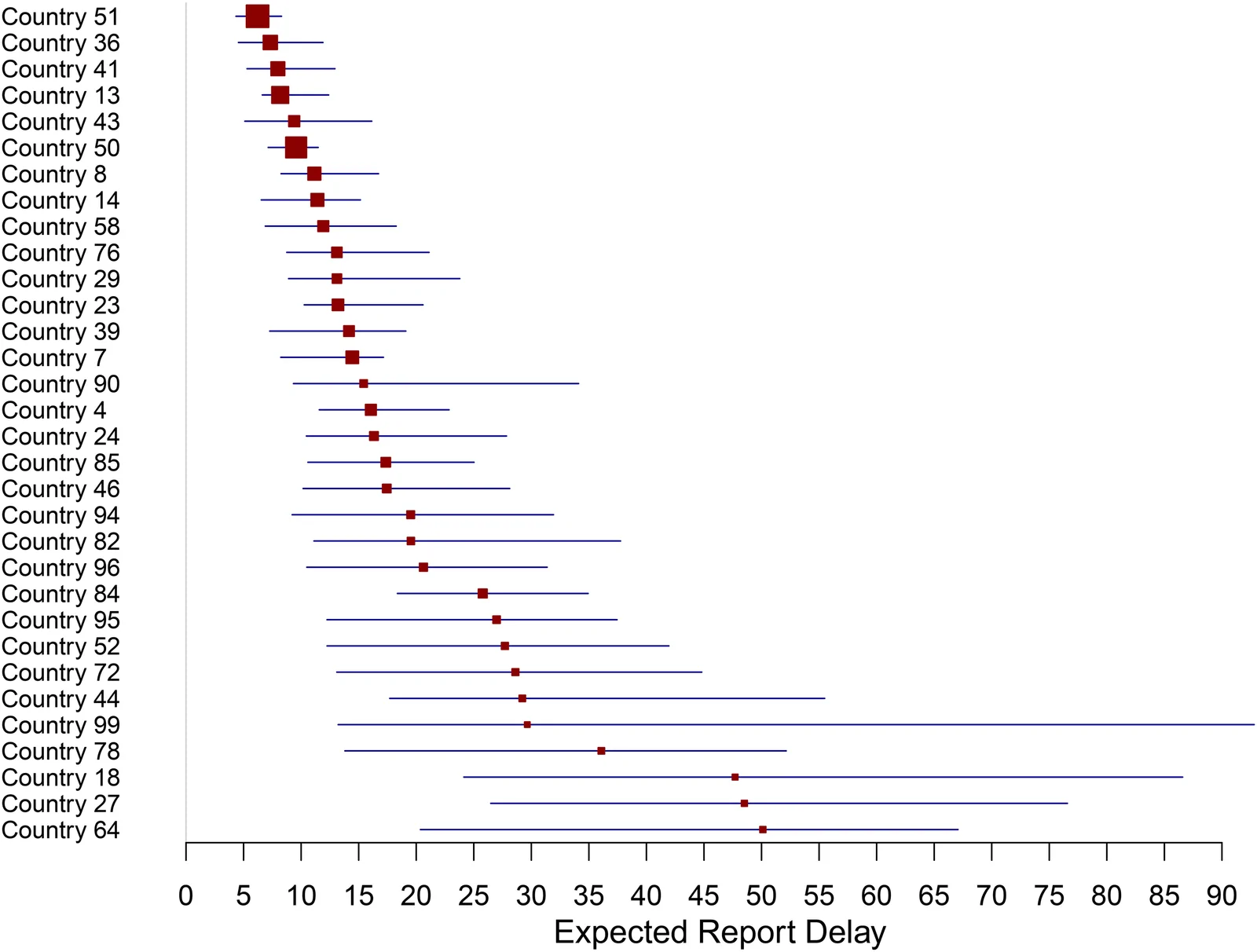
Expected delay in reporting HPAI outbreaks in various European countries. The expected report delay was calculated 1000 times per country, each time using a single randomly selected observed set of covariates from that country and a single randomly selected sample of parameters (obtained from the MCMC samples from the joint distribution). The mean and 95% CIs on those estimates of expected report delay are shown.

Considerable nonlinear temporal variation was detected by the P-spline (Fig 9), and indicated that the baseline hazard was negligible on day zero, rose to 0.28 (95% CI: 0.22, 0.32) on day 11, dropped to 0.13 (0.10, 0.16) on day 30, and stabilized at approximately 0.048 (0.036, 0.060) by day 240.

**Fig 9:**
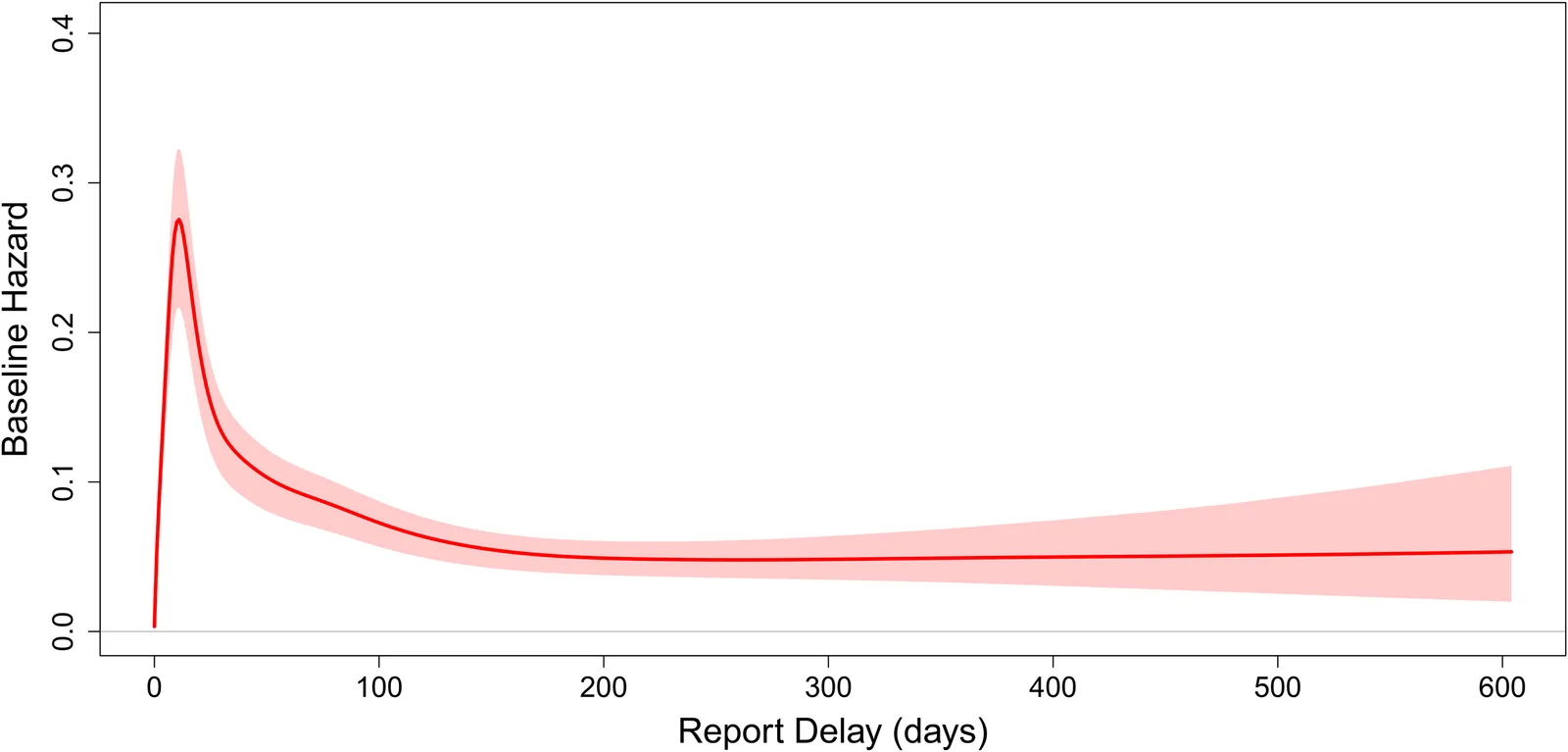
The effect of time (report delay) on the declaration rate (baseline hazard) of highly pathogenic avian influenza outbreaks in Europe. Non-linearity was inferred using a penalized spline. The expected value and the 95% CIs for the baseline hazard, calculated from the spline and intercept $\beta_{0}$, are shown in red and pink respectively.

### Other covariates

Several additional covariates were statistically related (indicated when a coefficient’s 95% CI excludes zero) to HPAI reporting delays (Fig 5, Table S2 in S1 File). Here we report the expected values and 95% CIs of the regression coefficients, plus the 95% CIs of the associated hazard ratios. Outbreaks in wild birds were associated with longer reporting times compared to those in domestic poultry (HR = 0.51, 95% CI: 0.49, 0.54). Countries with good “control of corruption” score according to the World Bank Worldwide Governance Indicators exhibited slower reporting times, the regression coefficient being −0.18 (95% CI: −0.34, −0.008) and 95% of hazard ratios ranging from 0.73 to 1.61 (Table S4 in S1 File). Similarly, countries with greater political stability showed slower reporting times, the regression coefficient being −0.17 (95% CI: −0.22, −0.11) and 95% of hazard ratios ranging from 0.77 to 1.72. However, countries with a high Gross National Income (GNI) per capita demonstrated shorter reporting delays, the coefficient being 0.24 (95% CI: 0.14, 0.34) and 95% of hazard ratio ranging from 0.61 to 1.59. Additionally, areas with high nearby poultry density were associated with shorter reporting delays, the coefficient being 0.076 (95% CI: 0.057, 0.093) and 95% of the hazard ratios ranging from 0.96 to 1.17. The recent HPAI incidence was also negatively affected reporting rates, with reporting taking approximately twice as long during high incidence periods than during low incidence periods (Table S4 in S1 File).

## Discussion

The current study investigated factors that potentially influence the timeliness of zoonotic infectious disease reporting in Europe, using Highly Pathogenic Avian Influenza (HPAI) as a model disease. We used the WAHIS dataset [12], consisting of 12166 reported HPAI outbreaks between January 2006 and May 2023, and a semi-parametric survival model, to evaluate the effects of socio-economic, technical capacity, epidemiological, and cultural covariates on the time taken to report outbreaks of HPAI. Since the suspected start date was used as the lower bound of each report delay, our study accounts for delays accumulated prior to laboratory testing, such as the time required to collect samples following the detection of clinical signs, as well as subsequent delays to notification. Whilst 74% of outbreaks were reported within one week, which is consistent with international standards requiring countries to submit follow-up reports on a weekly basis, 10% and 1% of outbreak reports took longer than 45 days 163 days respectively. Delays of this order clearly present barriers to the surveillance and control of HPAI spread in Europe.

Our results suggest several covariates may affect report delays, despite the use of Bayesian shrinkage to mitigate over-fitting. Some of the most striking effects included: large between-country differences; heterogeneity throughout the working week; and a non-linear effect of time (Fig 9). Other detected potential effects included: recent notification incidence; whether wild or domestic animals were involved; whether the event was a primary or secondary outbreak; and national holidays. Results highlighted the potential influence of country-specific factors, including: political stability; corruption control; Gross National Income (GNI); and poultry density. These findings underscore the multifaceted nature of report delays and the impacts of diverse epidemiological and socio-economic factors upon them.

Large between-country differences in reporting rates were observed. The expected report delay was less than ten days in six of the 32 countries; greater than 20 days in 11 countries; and greater than 35 days in four countries (Fig 8). This between-country variation is evident in the non-parametric summaries of the raw data (Fig 4), and was convincingly characterized by the semi-parametric model (Fig 7). The country effect coefficients (Fig 6) were large compared to other coefficients (Fig 5), although other covariates, including outbreak characteristics, economic factors, or governmental attributes, also helped characterize between-country heterogeneity. Fig 8 summarizes the expected distribution of report delays generated from 1000 simulations per country; each simulation used a randomly selected observed set of covariates from that country and a single set of model parameters sampled from the posterior distribution. This provides a comprehensive between-country comparison – combining country effects (Fig 6) with all additional sources of variation (e.g., incidence, political stability, etc) – of the expected report delays. Anonymisation of country names was a way to prevent discrimination, because identifying specific countries with longer notification delays could: i) lead to possible stigmatization that may not reflect current practices (as systems and practices may evolve over time); ii) create trade barriers, particularly given that notification delays can result from legitimate factors such as resource constraints, surveillance capacity, or geographic challenges rather than negligence, iii) discourage transparency in future reporting if countries being publicly named out. Comparing Figs 6 and 8 highlights that, while country-level coefficients explain much of the observed variation, additional contextual factors, including poultry density, whether the outbreak was in wild birds and socio-economic factors, also help characterize report delays across Europe. These findings emphasize that reducing report delays requires addressing not only country-level administrative and regulatory structures, but also factors linked to surveillance conditions during specific outbreaks, such as differences in monitoring capacity for wild birds, weekend and holiday effects, laboratory workload and saturation, and heterogeneity in surveillance and reporting practices across countries Targeted interventions, such as improving surveillance systems in high-risk settings or leveraging digital tools to streamline reporting processes, may help mitigate the most severe delays and improve overall outbreak response efficiency.

Another striking result relates to temporal variation. The penalized spline showed reporting rates rose rapidly during the 11 days following a suspicion, before dropping sharply, and then gradually, until stabilizing after 150 days. The non-linear drop is associated with a long-tail in the report delay distribution, and implies that once a report delay has grown beyond a certain duration predicting the submission date becomes difficult. Considerable day-to-day variation in reporting rates was also detected, with lower reporting rates during weekends and public holidays. Perhaps surprisingly, the national holiday effect was smaller than the weekend effect. Reporting rates were greatest on Fridays and lowest on Wednesdays. The Friday effect suggests that many laboratories or veterinary authorities strive to submit their reports prior to weekends. The Wednesday effect possibly arises from the fact that in some countries, Wednesday is a traditionally a non-school day for children, during which many professionals work reduced hours [43]. This within-week heterogeneity offers a potential area for reducing report delays, especially considering that only 44.9% of the 590 primary outbreaks were reported on the same day as the laboratory confirmation, as required by international standards. Similarly, the low reporting rates on Saturdays, Sundays and national holidays suggest that if, with suitable investment, staffing levels and operational capacities were increased during these periods, the timeliness and effectiveness of HPAI outbreak responses could be substantially enhanced.

Here, the national incidence of outbreak reports over the four weeks prior to the suspected outbreak start date, a proxy for the workload of disease surveillance teams, was negatively related to reporting rates. Similar findings have been described elsewhere [44]. This effect may be influenced by massive and punctual sample collections, organized in the framework of targeted or planned surveillance in some countries – such campaigns can generate large volumes of samples requiring analysis and positive results requiring reporting [45]. Current regulations may also be of influence. Primary cases should be reported within 24 hours of confirmation, according to Article 1.1.3 of the WOAH Terrestrial Animal Health Code [18]. These immediate notifications (INs) concern exceptional events, such as the first occurrence of a listed disease in a country or subnational area defined for reporting purposes in WAHIS, the re-emergence of an eradicated disease, or the emergence of a new strain. Subsequent weekly follow-up reports (FURs) provide further information on the evolution of events. As an epidemic grows new outbreaks are increasingly secondary and declared in FURs, which may partially explain why incidence appears to affect reporting rates. However, this explanation is incomplete: the FUR factor in the model didn’t eliminate the incidence effect; and 26% of outbreaks remained unreported after one week. The slower reporting of secondary outbreaks likely reflects less stringent reporting (to WAHIS) requirements for WOAH Members [18]. Whilst the WOAH stipulates general guidelines, the implementation can vary among member countries. The Terrestrial Animal Health Code specifies that weekly FURs should continue until the disease has been eliminated or the situation has become sufficiently stable that six-monthly reporting satisfies the obligation of member countries. However, the criteria for determining when a situation is “sufficiently stable” are not explicitly defined by WOAH, granting countries some discretion. This can lead to differences in reporting practices, including the proportions of INs versus FURs, and the intervals between successive FURs. For instance, some countries might opt to submit FURs more frequently during periods of peak outbreak activity, while others may adhere strictly to the weekly guideline. Additionally, the decision to cease weekly FURs and transition to less frequent reporting may be influenced by national policies, the perceived stability of an epidemic, and available resources. Understanding these differences is crucial for interpreting international reporting data and assessing the effectiveness of disease surveillance and control measures globally.

According to international regulations, the reporting of outbreaks in wild animals should follow the same rules as for domestic animals [18]. However, our results indicate longer reporting delays for outbreaks in wild birds: the mean reporting delay for domestic and wild birds being 15.5 (sd = 27.6) and 27.4 (sd = 36.4) respectively. This difference might be arise from the relatively low perceived economic impact of HPAI detection in wild birds or non-commercial holdings: the official HPAI status of a country is unaffected and there is no international trade ban (terrestrial code 10.4.1.3). Another explanation could be that longer confirmation delays arise due to logistical challenges in wild bird surveillance. Unlike the structured networks of poultry farms, where monitoring, sampling, and testing are well-integrated, wildlife surveillance is more opportunistic, relying on passive detection, sample transportation over greater distances, and more complex diagnostic workflows [46]. These procedural hurdles may slow outbreak confirmation. Despite these challenges, wild birds, particularly migratory waterfowl, play a key role in HPAI spread, facilitating long-distance dispersal and spillover to domestic poultry [47–49] – elements which emphasize the importance of timely detection and reporting. Accelerating the reporting of HPAI in wild birds would enable neighboring countries to enhance preparedness, particularly during migration periods [50]. Our findings highlight the need for improved surveillance strategies for wild birds, ensuring early detection and mitigating the risk of cross-species transmission.

High poultry density areas exhibited faster reporting. These areas often have more substantial biosecurity infrastructures due to increased economic stakes and higher disease transmission risk [51]. Such infrastructure includes better-trained staff, more frequent sampling, and advanced diagnostic tools. As a result, outbreaks in poultry-dense regions are detected and reported more quickly, leading to faster implementation of control measures. This surveillance efficiency helps mitigate spread within poultry, which reduces the risk of spread to other (domestic or wild) bird communities [52].

Our results suggest that countries with a high per capita GNI tend to report HPAI outbreaks more quickly. This is consistent with previous results highlighting a correlation between economic resources and the efficiency of disease surveillance systems [11,15]. Countries with a higher GNI often have more advanced healthcare infrastructures, better-funded research institutions, and more comprehensive public health policies, all of which facilitate faster detection and reporting of outbreaks. Studies have shown that wealthier nations are better equipped to implement robust surveillance mechanisms and respond quickly to emerging health threats [53]. Given the importance of rapid notification, enhancing the surveillance and reporting capacities of lower-GNI countries is essential for improving global preparedness and responses to HPAI outbreaks [54].

Results here revealed an intriguing association between political stability, corruption control and timely reporting. Perhaps counterintuitively, countries with greater political stability and better control of corruption exhibited longer reporting times for HPAI outbreaks. This suggests that political stability and low levels of corruption do not necessarily translate into more efficient disease reporting. One possible explanation could be the presence of bureaucratic processes that, while ensuring thoroughness and accuracy, may inadvertently delay outbreak reports [55]. Indeed, previous findings suggest that government transparency does not always correlate with improved efficiency in public health responses – [56] highlighted that transparent governance structures can sometimes lead to delays due to extensive verification and approval processes [56]. Nevertheless, the complex interplay between political stability, corruption control, and public health infrastructure underscores the need for a nuanced interpretation of how these factors influence disease reporting. Enhancing global preparedness for HPAI outbreaks requires addressing underlying systemic issues, and streamlining reporting processes, to ensure transparency and efficiency are balanced effectively.

Our study offers unique insights that could benefit existing European predictive HPAI risk models, such as Bird Flu Radar [57]. These models, which use environmental and migration data to predict disease introduction and establishment risk, heavily rely on outbreak reports to calibrate their predictions. However, these reports can be delayed by various socio-economic and cultural factors, such as national reporting behaviors and administrative structures. By analyzing these macroscopic cultural covariates, we bridge a critical gap that most existing models do not address – report delays can distort real-time predictions, making it essential to consider not only ecological risk factors, but also social and administrative barriers that influence the timeliness of outbreak data. This broader approach complements the spatial and environmental predictions provided by models such as Bird Flu Radar [57], and offers insights into the reasons behind reporting inconsistencies. Integrating cultural, administrative and socio-economic factors into predictive systems could significantly improve the ability to predict both outbreak locations and reporting rates. Such integration could strengthen early warning systems and reduce response times to HPAI outbreaks across Europe.

Although our study primarily focused on macroscopic covariates, several smaller-scale factors may still greatly influence outbreak reporting rates. Though less evident at a continental level, these parameters likely have regional or local relevance that should not be overlooked in future studies or when fine-tuning notification systems. First, the subtype or strain of the HPAI virus could affect notification rates. As mentioned, exceptional events, such as detecting a new or unusual strain, often trigger immediate notification. Therefore, subtypes representing novel introductions or mutations may prompt quicker responses than well-known strains. A subtype of particular interest – due to its potential to spread across borders – could see faster notifications as authorities aim to prevent broader epidemics [50]. Further research on strain-specific timelines could facilitate preparedness for future outbreaks and emerging zoonotic risks. In addition, the bird species involved in outbreaks may have nuanced effects on notification rates. Economically important species, for hunting or livestock purposes, can prompt varying levels of urgency in reporting. Waterfowl, such as ducks, are especially relevant here, as they are both migratory birds and known reservoirs of HPAI [49].

Beyond their economic relevance, the social importance of poultry should not be underestimated. Poultry play significant roles, both economically and socially, in many rural communities. Small-scale farmers might delay reporting outbreaks out of fear of culling and associated losses, while in areas where poultry is deeply integrated into social life, reporting may be swift due to local and community pressures to protect shared resources. Personal incentives for individuals involved in reporting, such as farmers, veterinarians, or public health officials, could introduce biases at local levels that are hard to detect in continental analyses. Investigating personal and social factors, such as fear of economic penalties or loss of livelihood, may provide insights in areas prone to under-reporting or delayed reporting [58].

Another important factor is the vaccination context at the time of the outbreak. Countries with robust vaccination programs may face fewer outbreaks, but this could lead to delays in reporting as there is a greater reliance on the perceived effectiveness of the vaccines. Additionally, vaccinated birds may show atypical symptoms, causing hesitations in confirming cases. Conversely, during vaccination campaigns, increased staff presence is required for both administration and enhanced surveillance, ensuring that potential outbreaks are detected early, even in vaccinated populations. This operational complexity may sometimes lead to delayed reporting as the focus shifts toward monitoring and verification [59,60]. In contrast, countries without vaccination programs may remain more vigilant, ensuring swift detection and reporting to prevent uncontrolled spread of the disease [61].

Lastly, the official status of the disease at the time of reporting could have a notable effect. Countries that have declared themselves free of HPAI can be more cautious in reporting new outbreaks, knowing that doing so would reverse their HPAI-free status and potentially trigger trade restrictions [62]. Such status-driven hesitation may result in delays, particularly in regions where the economic ramifications of losing the disease-free status are severe.

Various limitations of the current study should be considered when interpreting the results discussed above. First, there was considerable heterogeneity in the number of data points available for the different countries, and three countries had less than ten notifications included in the analysis (S6 in S1 File). This did not necessarily arise from lower incidence rates in those countries, but also reflects that complete sets of covariate data were sometimes not available, and thus some notifications were filtered from the analysis (S1 in S1 File). Whilst this choice had the potential to lead to large uncertainties in the estimated notification rates of those countries, the use of Bayesian shrinkage would have helped mitigate these effects. Moreover, the 95% credibility intervals of the hazard ratios in those countries contained the value one, suggesting that data sparsity did not lead to systematic bias in the hazard ratio estimates for those countries. A second potential limitation here was that we did not explore the sensitivity of the results to the choice of hyperparameter in the Bayesian shrinkage. Bhattacharya *et al* [29] explored setting the shape parameter to the inverse of the number of covariates, but reported that this leads to over-shrinkage when effect sizes are small. Thus, we adopted their protocol of setting the shape to 1/2. Whilst we could have placed a prior on that parameter, it would have greatly increased computation time and was therefore considered beyond the scope of the current study. Thirdly, a similar limitation arises in the choice of penalization of the P-spline. In our implementation τ was fixed at a value that allowed considerable non-linearity to be detected in the data, whilst avoiding visible signs of over-fitting. An alternative approach would have been to place a prior on τ, however, this would have not necessarily removed a potential source of subjectivity, and would have considerably increased computation time.

## Conclusion

Here, WAHIS data were used to investigate how report delays for HPAI outbreaks in Europe are affected by various socio-demographic, technical, epidemiological, geographical, and cultural factors. Results indicate large space-time heterogeneity in reporting rates, including substantial between-country differences. Key covariates identified here point to actionable strategies for reducing report delays, such as improving international harmonization of surveillance capacities, providing targeted incentives, and enhancing the processing of outbreaks in wild birds. Further research is needed to refine understanding of how disease report delays are affected by various factors, including the economic dimensions of the poultry industry and local reporting practices. Incorporating systematic delay adjustments into disease forecasting models could enhance epidemic predictions, particularly for wildlife outbreaks where reporting lags may distort perceived trends. The transferability of these findings to other zoonotic and animal diseases listed by WOAH remains uncertain. HPAI benefits from comparatively high surveillance sensitivity, well-established diagnostic pathways, and frequent notifications, which may limit the direct applicability of quantitative results to diseases with lower reporting intensity or less developed surveillance infrastructure. Nonetheless, the modelling framework presented here is general and can be applied to other WAHIS datasets, which could facilitate future cross-disease comparative studies and methodological extensions. The results presented here provide a foundation for improving surveillance systems and ensuring more timely responses to future outbreaks of animal diseases.

## Supporting information

S1 File S1 Data. Curation and Covariate Selection. S2 Data. Additional results. S3 Data. Model Outputs.
(PDF)
