# Supplementary material for: Factors influencing the timeliness of reporting highly pathogenic avian influenza outbreaks in Europe
Source: PLoS One. 2026 Nov 10;21(11):e0360917. doi: 10.1371/journal.pone.0360917 (PMC13750615; doi:10.1371/journal.pone.0360917)
Supplement: Supplementary file 1 [file pone.0360917.s001.pdf]

Supplementary Material

S1 Data Curation and Covariate Selection

S1A Curation Process

The process of curating data for the analysis of HPAI report delays in Europe is presented in figure S1.

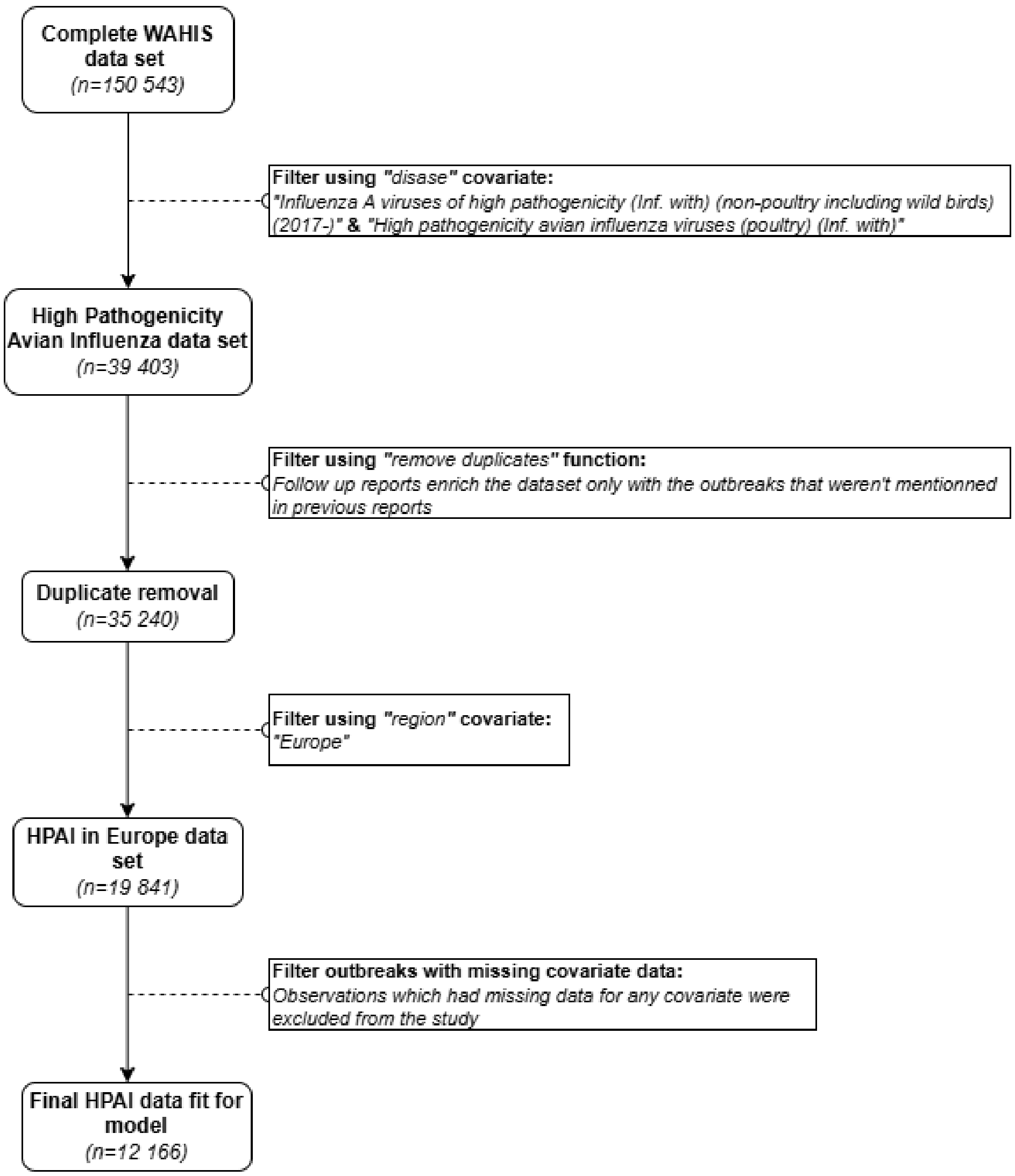

Fig S1. Data curation flow chart

S1B Motivation for the Covariates

The following section provides a brief motivation behind the choice of each covariate:

- **Wild/Domestic:** Previous studies suggest that wild outbreaks tend to be reported with greater delay compared to domestic outbreaks [37]. Wild hosts may be more challenging to detect and confirm than domestic ones, given their less controlled environments and the difficulty in obtaining samples, potentially leading to longer reporting times. In addition, we assume that an outbreak detected in poultry might be reported immediately due to economic consequences and exports regulation.

- **Year of First Confirmation:** The time elapsed since the first recorded case of HPAI in a country may influence reporting efficiency, as countries with longer exposure to the disease may have improved their surveillance and response systems over time. Conversely, newly affected countries may face delays due to lack of experience or preparedness.
- **Incidence:** The number of reports submitted in the two weeks preceding the notification date can reflect the intensity of an ongoing outbreak. High incidence rates may lead to quicker reporting due to heightened surveillance, or conversely, overwhelm reporting systems and cause delays.
- **Primary/Secondary Outbreak:** Primary outbreaks may be reported more promptly than secondary outbreaks, as primary outbreaks often trigger a rapid response from health authorities. Secondary outbreaks might be identified through tracing or secondary transmission, potentially leading to reporting delays.
- **Poultry Exports:** Countries with higher live poultry exports may have more stringent and faster reporting systems in place due to the economic importance of their poultry industries. The pressure to maintain international trade standards could incentivize faster reporting of outbreaks.
- **Local Poultry Density:** Areas with higher densities of ducks and chickens may experience either quicker reporting due to heightened surveillance or delayed reporting due to the difficulty of managing and monitoring large populations.
- **Veterinarians per Capita:** A higher number of veterinarians relative to the population may indicate stronger veterinary infrastructure, which could lead to quicker detection, diagnosis, and reporting of outbreaks.
- **State Structure:** The organizational structure of a country's government (federal vs. centralized) may affect the efficiency of communication and response times. Centralized systems may have more streamlined reporting channels, while federal systems could face delays due to decentralized decision-making [24].
- **Financial Capacity (GNI & GDP):** Gross National Income (GNI) and Gross Domestic Product (GDP) is used as a proxy for the financial capacity of a country, with the hypothesis that nations with higher GNI possess greater resources to allocate towards their animal health infrastructure. This may lead to more efficient detection, diagnosis, and reporting of outbreaks. Previous research has indicated that economic factors such as GNI play a role in shaping the timeliness of health surveillance and response systems in both human and animal health contexts [37]. In the present study, GNI is integrated into a multivariable model to explore its impact alongside other factors affecting reporting delays, broadening the understanding of its role in the dynamics of zoonotic disease surveillance.
- **Government Stability (Stability and Control of corruption Scores):** Stable governments with low corruption are more likely to have well-functioning, transparent reporting systems. Instability or high corruption could impede the timely reporting of outbreaks due to inefficiencies or lack of accountability.
- **Communication Device Usage (Internet and Phone Usage):** Greater internet and phone penetration among the population may facilitate quicker dissemination of outbreak information and improve coordination within the animal health network, leading to more timely reporting.
- **Day of Week and Holidays:** Reporting may be affected by operational schedules, with lower reporting rates expected during weekends and public holidays when health authorities and laboratories may not operate at full capacity. This covariate is important to account for cultural and operational delays in reporting.
- **Day-Zero Effect:** The day of PCR confirmation may have fewer working hours available for report submission compared to subsequent days, leading to potential delays in reporting. This factor accounts for the operational limitations on the day of diagnosis.

S1C Candidate Covariates Evaluation

Initial Selection An initial set of 18 candidate covariates was selected based on epidemiological, socio-economic, and environmental factors. However, two variables were removed at the outset: Veterinary capacity, due to insufficient data quality, and State structure, which was considered too arbitrary to measure consistently across different countries.

Cross-Correlation Analysis To mitigate issues of multicollinearity and redundancy, a cross-correlation analysis was performed on the remaining covariates (Fig. S2). This analysis led to the removal of 4 covariates that exhibited high c orrelations (Pearson's $-0.8 > r > 0.8$) with others: First, GDP per capita was highly correlated with GNI per capita (correlation coefficient = 0.963), and was thus excluded in favor of GNI, a more stable measure. Similarly, the "Control of corruption score" showed a high correlation with both GDP per capita (0.867) and GNI per capita (0.896), leading to its removal to avoid excessive multicollinearity.

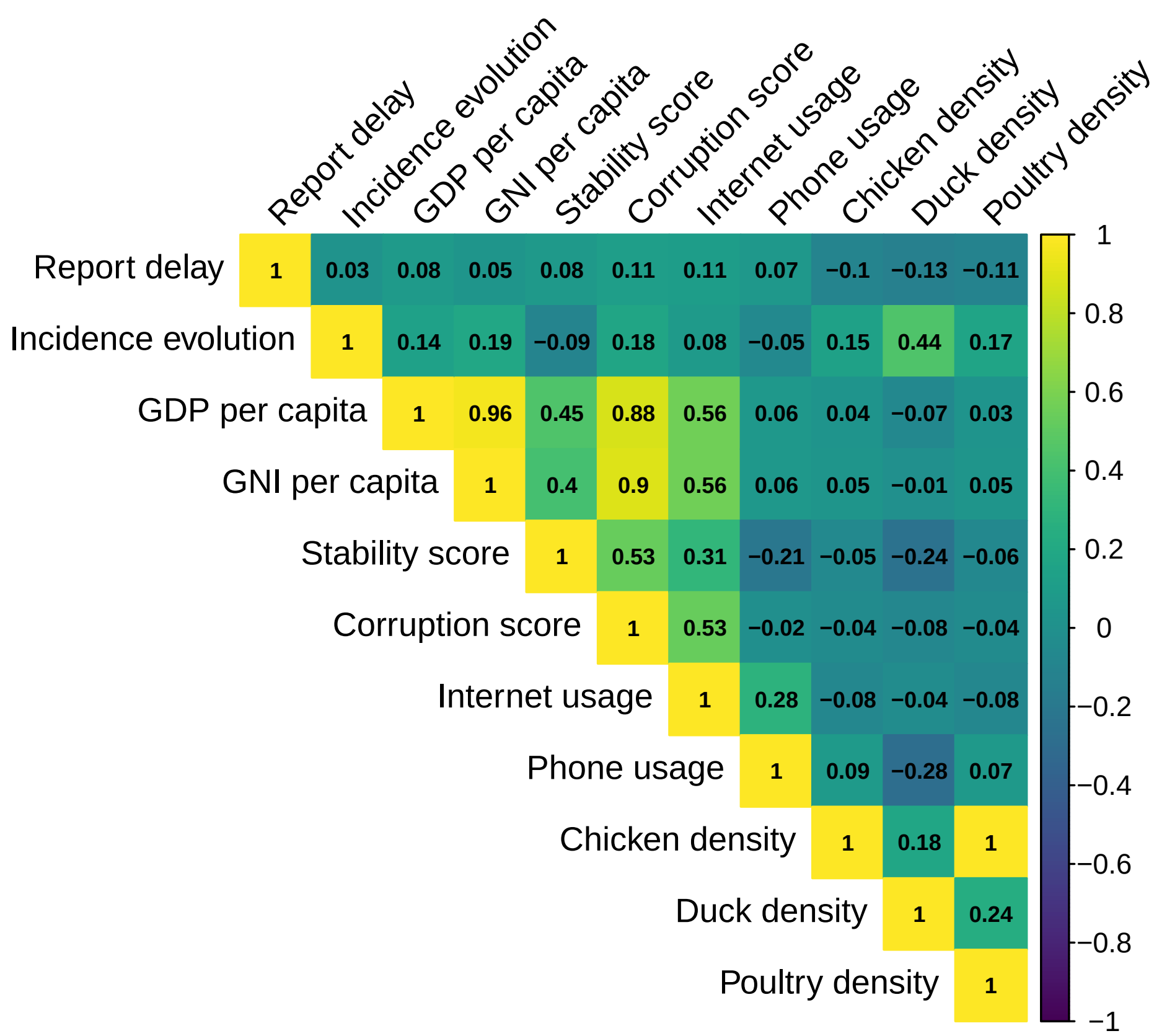

Fig S2. Cross-correlation heatmap for initial candidate covariates. "GDP per capita" also has high (0.96) correlation with "GNI per capita". Additionally, "Control of corruption score" has high correlation with "GDP per capita" (0.88) and with "GNI per capita" (0.9).

Additionally, although chicken density and duck density provide useful measures of bird population, they were excluded due to their strong correlation with poultry density. Chicken density was highly correlated with poultry density (0.998), while duck density showed a moderate correlation (0.247) but was nonetheless excluded to simplify the analysis and avoid overlapping metrics, as poultry density serves as a more comprehensive variable.

Variance Inflation Factor (VIF) Analysis A Variance Inflation Factor (VIF) analysis was conducted to further assess multicollinearity (Fig. S3). While no covariates displayed excessively high VIF values that

would typically warrant exclusion (cutoffs of 5 or 10 are commonly used) [13], this check was performed to ensure robustness and avoid potential collinearity issues in the final model.

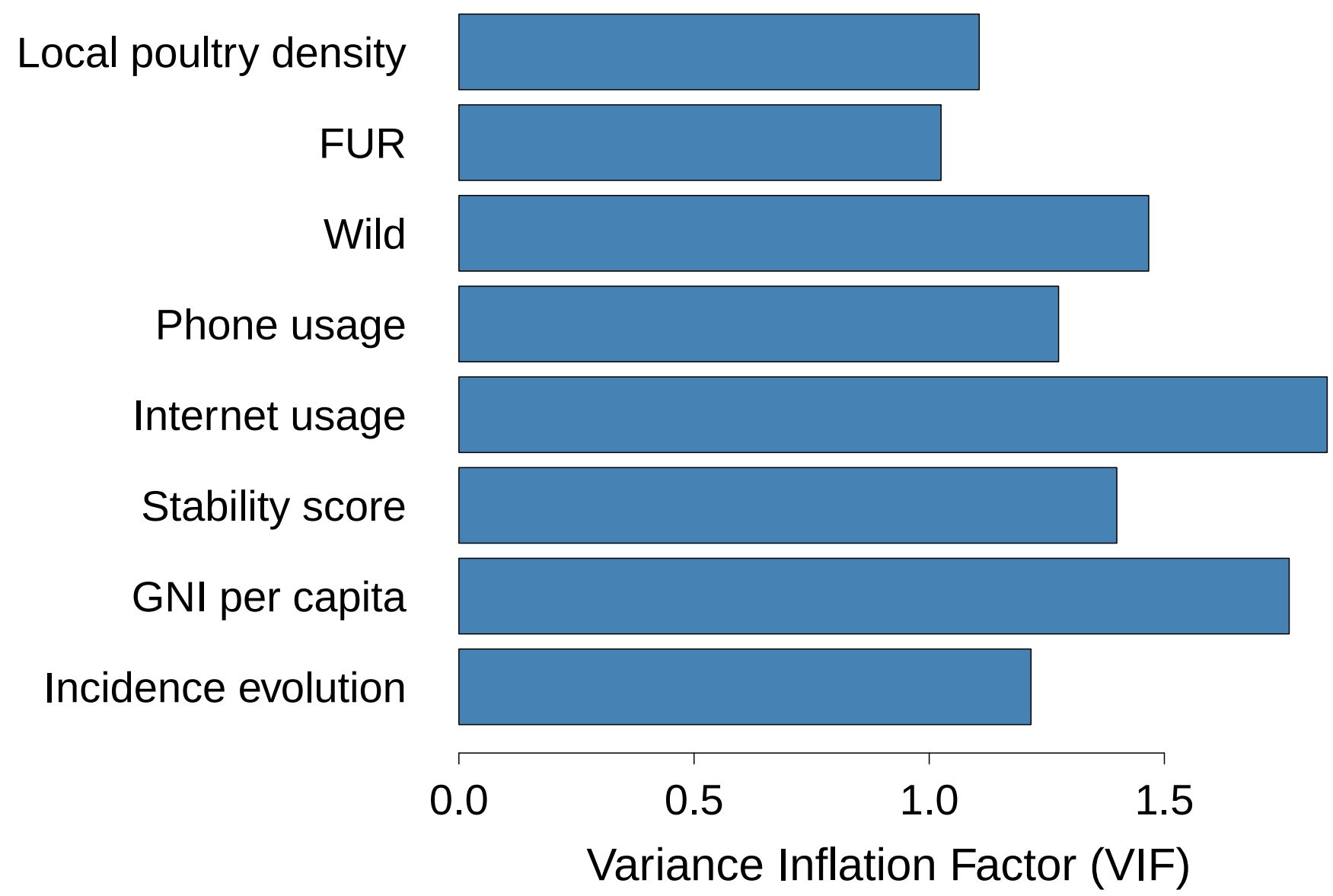

Fig S3. Variable Inflation Factor (VIF) of candidate covariates after the removal of overly correlated covariates. The maximum value for the VIF is "Internet usage" = 1.850.

S2 Additional results

S2A Cross tabulation of lab confirmation dates and reporting date

A cross tabulation of week days for each pair of confirmation date and report date in the HPAI report delay data is presented in Table S1 (only available for immediate notifications).

	Monday	Tuesday	Wednesday	Thursday	Friday	Saturday	Sunday	Sum
Monday	6	**34**	11	15	11	0	0	77
Tuesday	8	7	**37**	25	9	3	2	91
Wednesday	15	4	12	**48**	21	0	1	101
Thursday	40	9	10	17	**45**	2	0	123
Friday	13	19	30	22	**39**	7	2	132
Saturday	**15**	9	1	3	1	2	1	32
Sunday	9	**20**	4	0	0	0	1	34
Sum	106	102	105	130	126	14	7	590

Table S1. Cross tabulated weekdays for each pair of confirmation date and report data in the HPAI report delay data. Rows indicate the day of the week on which a lab confirms an HPAI outbreak. Columns indicate the day of week on which the lab reports an HPAI outbreak. For highest number of each row is represented in bold.

S2B Non-parametric survival curves of the HPAI reports data

A non-parametric survival curve generated from the interval censored HPAI report delay data is presented in figure S4.

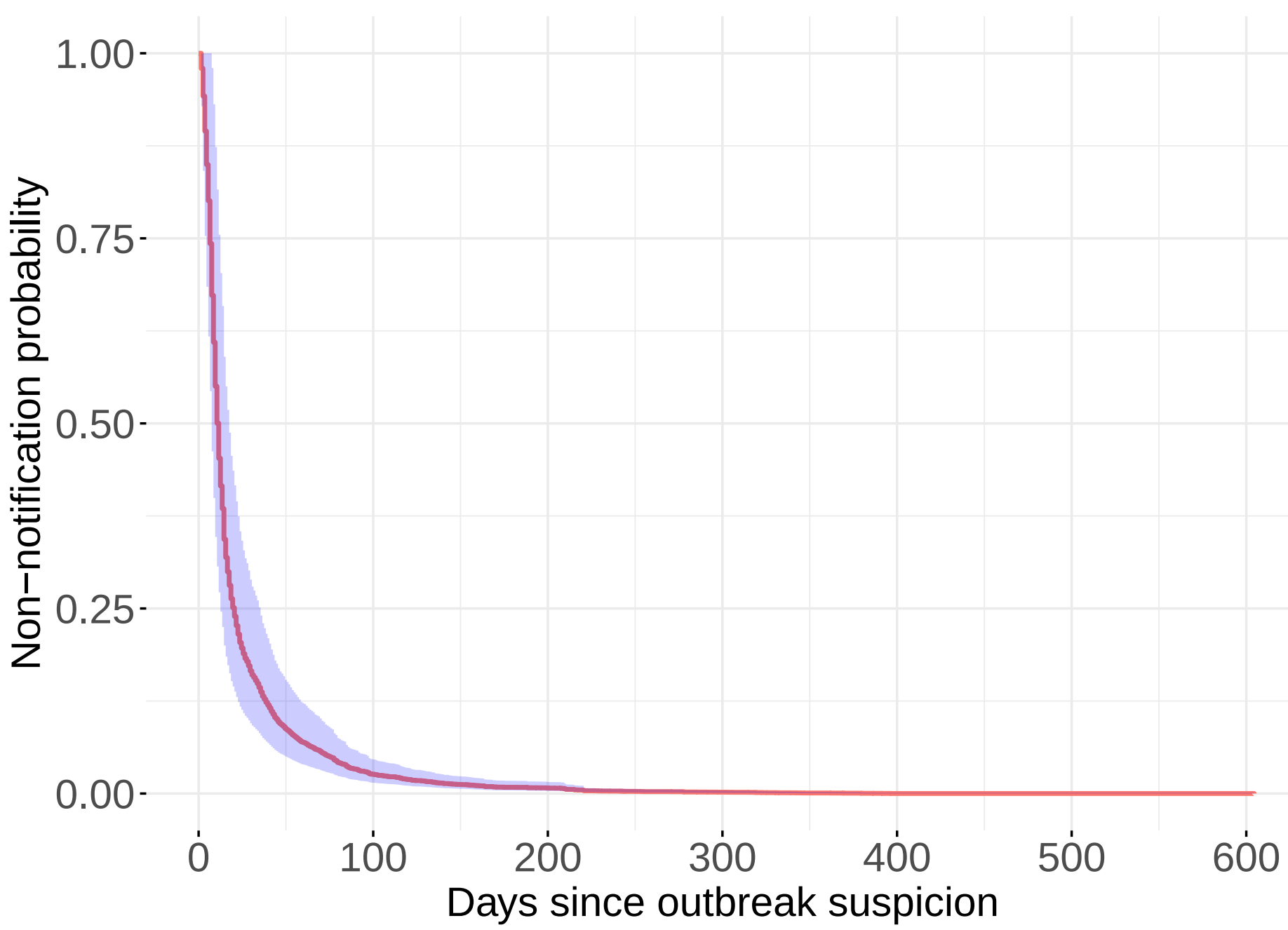

Fig S4. Non-parametric survival curve generated from the (interval censored) HPAI report delay data (red). The blue area represents the 99% confidence interval.

S3 Model Outputs

Here we present a series of tables and figures generated from output of the semi-parametric survival models used to analyze HPAI outbreak report delays.

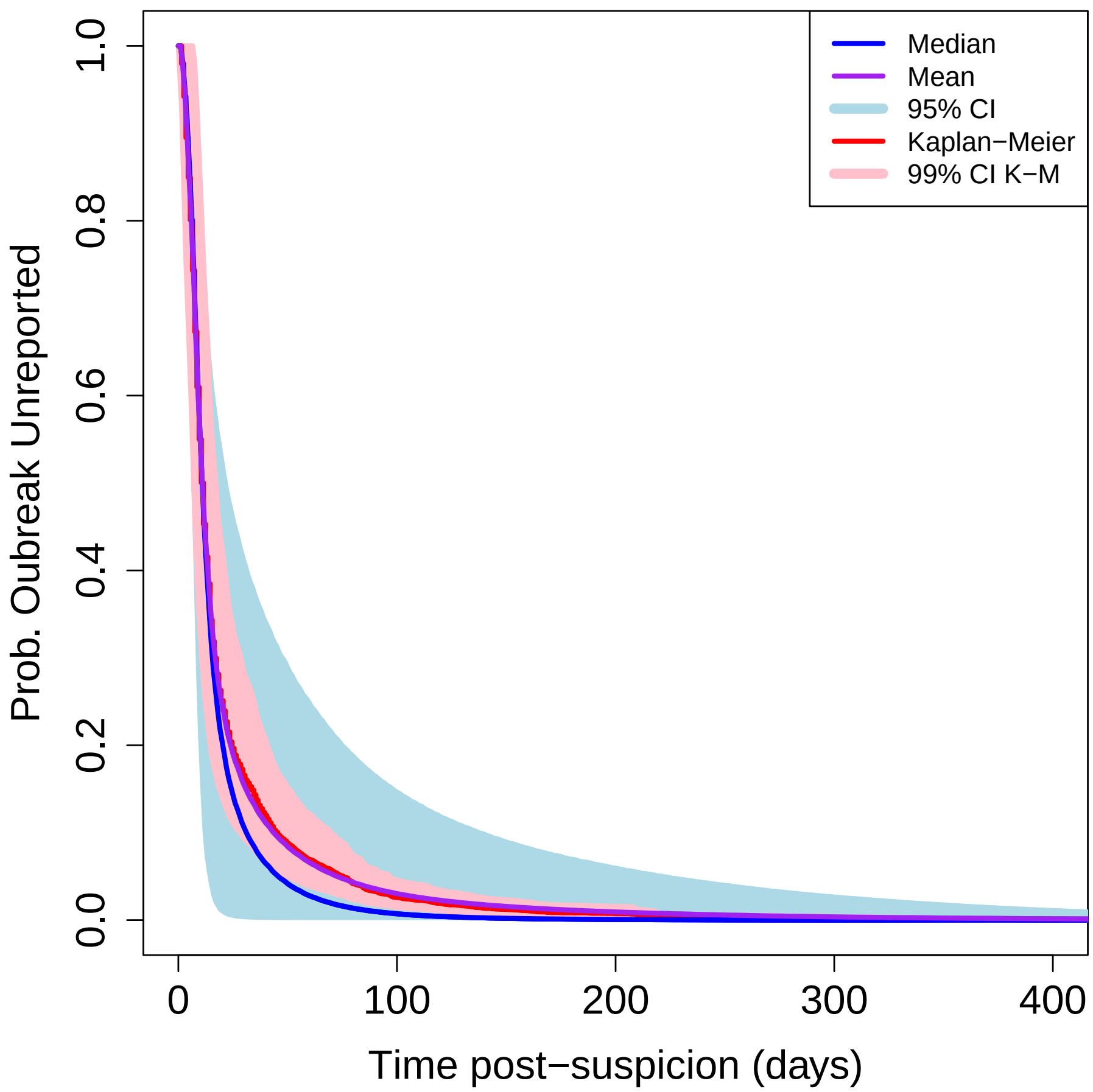

Fig S5. "Survival curves describing how the probability that an outbreak remains unreported decreases with time. The non-parametric survival curve (red) was computed directly from the observed reporting delays only, without incorporating any predictors. The expected (purple) and median (blue) survival curves correspond to predictions from our Bayesian model, which uses all covariates. Credibility intervals for the Bayesian semi-parametric model and for the non-parametric curve are shown in light blue and pink, respectively.

	Mean	2.5%	97.5%
Wild	-6.72E-01	-7.21E-01	-6.23E-01
Evolution_incidence_4weeks	-1.69E-01	-1.92E-01	-1.47E-01
GNI_per_Capita	2.44E-01	1.35E-01	3.42E-01
Stability_score	-1.67E-01	-2.23E-01	-1.10E-01
Control_of_corruption	-1.75E-01	-3.41E-01	-8.16E-03
Internet_usage	4.60E-02	2.15E-04	9.32E-02
Phone_usage	6.18E-02	7.83E-04	1.19E-01
Poultry_density	7.55E-02	5.70E-02	9.31E-02
FUR	-1.20E-01	-2.05E-01	-2.83E-02
Day_0	-5.48E+00	-1.31E+01	-1.58E+00
Monday	1.85E-02	-6.89E-02	1.89E-01
Tueday	4.00E-02	-3.95E-02	2.14E-01
Wednesday	-2.50E-01	-3.47E-01	-7.73E-02
Thurday	-3.04E-02	-1.26E-01	1.32E-01
Friday	6.60E-01	5.71E-01	8.26E-01
Saturday	-1.83E+00	-1.97E+00	-1.65E+00
Sunday	-2.12E+00	-2.27E+00	-1.92E+00
Holiday	-7.82E-01	-9.08E-01	-6.55E-01
beta0	-6.14E+00	-7.38E+00	-4.92E+00

Table S2. Posterior mean and 95% credibility intervals of coefficients (β) for covariates included in a semi-parametric survival model used to analyze report delays for HPAI outbreaks in Europe and neighboring countries. Country effects are omitted here for brevity, but are presented in figure 6.

	Mean	2.5%	97.5%
Country 51	4.15	2.74	5.90
Country 50	2.29	1.72	3.01
Country 14	1.59	1.17	2.12
Country 43	1.41	0.76	3.16
Country 85	1.20	0.87	1.87
Country 4	1.18	0.96	1.55
Country 41	1.16	0.98	1.44
Country 8	1.09	0.93	1.37
Country 13	1.09	0.88	1.55
Country 76	1.08	0.89	1.44
Country 36	1.07	0.78	1.59
Country 39	1.04	0.89	1.33
Country 7	1.01	0.86	1.24
Country 46	1.00	0.85	1.18
Country 58	0.97	0.73	1.21
Country 96	0.94	0.64	1.19
Country 29	0.91	0.46	1.31
Country 23	0.90	0.71	1.05
Country 99	0.85	0.27	1.40
Country 94	0.75	0.56	1.01
Country 90	0.67	0.34	1.03
Country 24	0.64	0.55	0.77
Country 72	0.59	0.50	0.75
Country 78	0.55	0.45	0.69
Country 52	0.50	0.35	0.73
Country 95	0.49	0.41	0.59
Country 44	0.45	0.38	0.55
Country 64	0.39	0.31	0.53
Country 84	0.37	0.29	0.49
Country 27	0.32	0.25	0.41
Country 82	0.25	0.18	0.37
Country 18	0.25	0.17	0.35

Table S3. Mean and 95% credibility intervals for the hazard ratios associated with country effects. Hazard ratios greater (lesser) than one imply faster (slower) than average reporting (respectively).

	0.5%	2.5%	25%	50%	75%	97.5%	99.5%
Evolution_incidence_4weeks	0.4759	0.5699	0.9411	1.0593	1.1191	1.1608	1.1702
GNI_per_Capita	0.5478	0.6060	0.8420	1.0649	1.1595	1.5537	2.0597
Stability_score	0.6941	0.7711	0.9058	0.9830	1.0953	1.6006	2.0332
Control_of_corruption	0.6611	0.7399	0.8847	0.9717	1.1028	1.5870	2.0019
Internet_usage	0.7149	0.8850	0.9963	1.0028	1.0247	1.0707	1.1041
Phone_usage	0.7871	0.8718	0.9830	0.9957	1.0308	1.1418	1.3296
Poultry_density	0.9609	0.9640	0.9709	0.9771	0.9994	1.1973	1.7364

Table S4. Effects of continuous covariates, represented as quantiles of 10000 simulated values of the hazard ratios. For each simulation a single line of MCMC output was selected at random to set the coefficients, and data from a single observation was also selected at random. The hazard ratio is simply the exponential of the product of the selected data and coefficient. In each case the expected value (not shown) is negligibly close one, because the data were centered. These hazards ratios can be compared with those generated from factors in Table S5.

	Mean	2.5%	97.5%
Wild	0.511	0.486	0.537
FUR	0.887	0.815	0.972
Day_0	0.032	0.000	0.206
Monday	1.021	0.933	1.208
Tueday	1.043	0.961	1.238
Wednesday	0.780	0.707	0.926
Thurday	0.972	0.881	1.141
Friday	1.938	1.771	2.285
Saturday	0.160	0.139	0.192
Sunday	0.121	0.103	0.146
Holiday	0.459	0.404	0.519

Table S5. Mean and 95% credibility intervals for the hazard ratios associated with factors. Hazard ratios greater (lesser) than one imply faster (slower) than average reporting (respectively).

	Mean	2.5%	97.5%	N
Country 51	6.21	4.35	8.24	29
Country 36	7.34	4.56	12.01	39
Country 41	7.95	5.34	12.64	548
Country 13	8.16	6.63	12.29	109
Country 43	9.41	5.15	15.99	5
Country 50	9.56	7.15	11.46	70
Country 8	11.15	8.25	16.68	320
Country 14	11.39	6.55	15.20	83
Country 58	11.93	6.88	18.12	32
Country 76	13.05	8.74	20.99	47
Country 23	13.20	10.27	20.47	1018
Country 29	13.25	9.00	24.55	6
Country 39	14.25	7.40	19.00	757
Country 7	14.44	8.23	17.12	288
Country 90	15.54	9.33	34.33	13
Country 4	16.04	11.62	22.75	76
Country 24	16.22	10.48	27.68	3165
Country 85	17.29	10.56	25.24	18
Country 46	17.41	10.15	28.17	198
Country 82	19.34	11.15	37.66	360
Country 94	19.57	9.22	31.92	74
Country 96	20.62	10.46	31.34	29
Country 84	25.75	18.38	35.20	149
Country 95	27.02	12.34	37.40	199
Country 52	27.73	12.31	41.95	60
Country 72	28.84	13.33	44.83	2619
Country 44	29.23	17.81	55.29	630
Country 99	29.70	13.01	93.46	2
Country 78	36.20	13.99	52.32	600
Country 18	48.12	24.50	86.70	44
Country 27	48.56	26.49	76.84	104
Country 64	50.13	20.32	67.17	475

Table S6. Expected report delay per country, represented as the mean and 95% credibility intervals obtained from 10000 model simulations per country. The number of HPAI outbreak reports over the study period is presented as N.
